# Solvothermally Grown Oriented WO_3_ Nanoflakes for the Photocatalytic Degradation of Pharmaceuticals in a Flow Reactor

**DOI:** 10.3390/nano14100860

**Published:** 2024-05-15

**Authors:** Mirco Cescon, Claudia Stevanin, Matteo Ardit, Michele Orlandi, Annalisa Martucci, Tatiana Chenet, Luisa Pasti, Stefano Caramori, Vito Cristino

**Affiliations:** 1 Department of Chemical, Pharmaceutical and Agricultural Sciences, University of Ferrara, Via L. Borsari 46, 44121 Ferrara, Italy; mirco.cescon@unife.it (M.C.); vito.cristino@unife.it (V.C.); 2 Department of Environmental and Prevention Sciences, University of Ferrara, Via L. Borsari 46, 44121 Ferrara, Italy; claudia.stevanin@unife.it (C.S.); tatiana.chenet@unife.it (T.C.); 3 Department of Geosciences, University of Padova, Via Gradenigo 6, 35131 Padova, Italy; matteo.ardit@unipd.it; 4 Department of Physics and Earth Sciences, University of Ferrara, Via Saragat 1, 44121 Ferrara, Italy; annalisa.martucci@unife.it; 5 Department of Physics, University of Trento, Via Sommarive 14, 38123 Trento, Italy; michele.orlandi@unitn.it; 6 National Interuniversity Consortium of Materials Science and Technology (INSTM), University of Ferrara Research Unit, 44121 Ferrara, Italy

**Keywords:** pharmaceuticals, photocatalytic prototype system, solvothermally grown WO_3_, water remediation

## Abstract

Contamination by pharmaceuticals adversely affects the quality of natural water, causing environmental and health concerns. In this study, target drugs (oxazepam, OZ, 17-α-ethinylestradiol, EE2, and drospirenone, DRO), which have been extensively detected in the effluents of WWTPs over the past decades, were selected. We report here a new photoactive system, operating under visible light, capable of degrading EE2, OZ and DRO in water. The photocatalytic system comprised glass spheres coated with nanostructured, solvothermally treated WO_3_ that improves the ease of handling of the photocatalyst and allows for the implementation of a continuous flow process. The photocatalytic system based on solvothermal WO_3_ shows much better results in terms of photocurrent generation and photocatalyst stability with respect to state-of-the-art WO_3_ nanoparticles. Results herein obtained demonstrate that the proposed flow system is a promising prototype for enhanced contaminant degradation exploiting advanced oxidation processes.

## 1. Introduction

**Figure 1:**
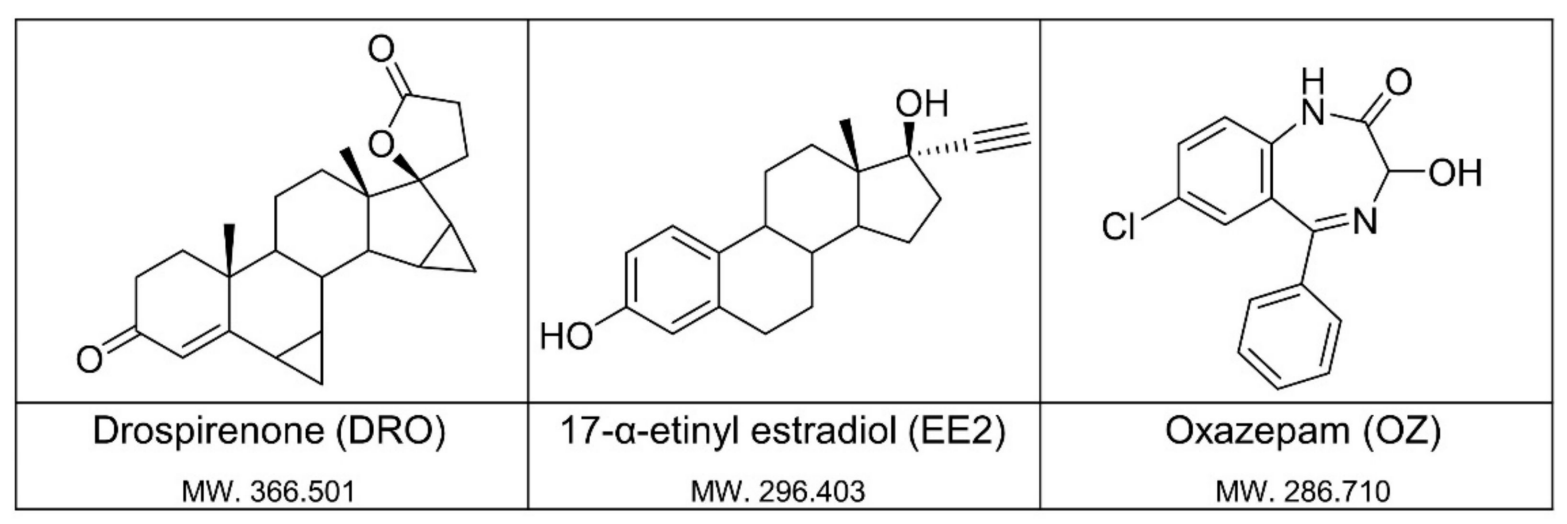
Molecular structure of drospirenone (DRO), 17-α-ethynilestradiol (EE2) and oxazepam (OZ). The molecules are all represented in their neutral form.

In recent years, a significant influx of new contaminants has adversely affected the quality of both surface and groundwater on a global scale. The increase in human life expectancy, coupled with population growth, has led to an escalation in the use of pharmaceuticals [1,2]. Consequently, this has enhanced the presence of drugs in aquatic environments, resulting in a comprehensive list that undergoes continuous updates. The growing reports of the detection of these contaminants in surface waters worldwide is a cause for concern. Their presence in the aquatic environment can induce alterations in the physiological conditions and behavior of aquatic organisms. In particular, among bioactive environmental contaminants, steroid hormones are considered of high priority due to their biological activity, widespread use, and potential to interfere in endocrine functions (e.g., reproductive dysfunction) after exposure [3,4]. Drospirenone (DRO) (Figure 1), a synthetic progestin used in oral contraceptives, is frequently combined with 17-α-ethinylestradiol (EE2) (Figure 1) [5]. It is one of the most prescribed synthetic progestins in numerous European countries and it is extensively used, especially in the USA. Due to their extensive use, both molecules have been detected in wastewaters and surface waters [6]. It has been demonstrated that fish species, such as adults and embryos of zebrafish (Danio rerio), exposed to DRO showed alteration in the gene transcription involved in hormone homeostasis, leading to reproductive changes and significant alterations of circadian rhythm [6,7]. Similarly, in surface waters, the synthetic estrogen EE2 can be found as a ubiquitous contaminant; indeed, EE2 at environmentally relevant concentration levels presents adverse effects on fish, including decreased egg fertilization, reproduction disruption, feminization, induction of vitellogenin, and increased mortality [8]. Due to its potential adverse effect on biota and human health, a similar estrogen (17β-estradiol) has been listed in Japanese Drinking Water Quality Standard [9]. In fact, EE2 considered as a priority micropollutant, as suggested by the European Union, might be regulated in the Drinking Water Quality Standards of European countries in the future [8].

Recent research reports that municipal wastewater treatment plants (WWTPs) are the main sources of EE2 and DRO [6,8] in effluents, and consequently, in surface waters, highlighting the importance of enhancing pharmaceutical removal performance of municipal WWTPs. Concentrations of DRO and EE2 in surface waters evaluated in many countries were found up to 4.3 ng L^−1^ and 21.5 ng L^−1^, respectively [9,10,11]. For what concerns psychoactive substances, oxazepam (OZ) (Figure 1) belongs to a family of pharmaceuticals called the benzodiazepines. In European river water, oxazepam is the benzodiazepine that is detected with the highest frequency (85%), with a maximum concentration of 61 ng L^−1^ [12]. Close to the outlet of WWTPs, OZ concentrations can reach several hundreds of ng L^−1^ to low ng L^−1^ concentrations, depending on the size of the receiving river and the season [13,14]. Psychoactive substances can raise behavioral alterations that may lead to profound, nonlinear, and perhaps unpredictable ecological effects [15]. One of the main sources of the mentioned contaminants are the effluents of WWTPs; therefore, it is important to develop innovative technologies to efficiently remove those contaminants from water in order to avoid the adverse effects on water and prevent irreversible damage to the aquatic ecosystems. Nowadays, different approaches for the removal of emergent contaminants (ECs) are employed, such as the use of adsorbent materials and advanced oxidation processes (AOPs) [14]. AOPs can be used as a pre-treatment [16,17] process to improve wastewater quality and remove organic contaminants by oxidation through reactions with hydroxyl radicals (OHs) which, being a strong oxidant characterized by an oxidation potential equal to E° = +2.1 V vs. NHE, allows for the oxidation of a variety of organic substances, leading to their mineralization [18,19,20]. To reduce the energetic request for implementing redox remediation processes, photocatalytic materials show some promise. 

The ability of some semiconductors to adsorb UV–vis photons for the production of highly energetic electron/hole pairs makes these materials capable of promoting thermodynamically demanding oxidation reactions. In particular, some metal oxide semiconductors like TiO_2_, ZnO and WO_3_ display such a deep valence band, whose edge bears the prevailing contributions by π orbitals of the oxygen atoms, that holes therein generated are able to produce hydroxyl radicals following monoelectronic water oxidation. The wide band gap of many metal oxide semiconductors can limit the portion of absorbed solar light; for example, TiO_2_ and ZnO have a sizable photochemical activity only upon UV excitation. A good compromise between the need for generating powerfully oxidizing holes and the harvesting of solar light is incorporated in WO_3_, being able to absorb photons up to 460 nm (band gap of ca. 2.7 eV). The ability of WO_3_ to generate ·OH through water oxidation under visible illumination has been previously reported [21]. A variety of techniques can be used to synthesize efficient WO_3_ structures aimed at increasing the photon-to-electron conversion by emphasizing the surface (contact) area of the photocatalyst, thus reducing electron/hole recombinative losses; pulsed laser deposition [22], electrochemical anodization [23,24,25,26], sputtering [27], evaporation [28], sol-gel [29,30,31], electrophoresis [32], hydrothermal and solvothermal [33,34,35] are some of the techniques described in the scientific literature. 

Besides achieving a high photoconversion efficiency, the choice of the preparative technique to afford a practical photocatalytic system for water decontamination should consider other aspects as well. The low cost and abundancy of reagents and of preparative equipment, the potential for scaling-up the synthesis, the possibility to support the catalytic material to facilitate the handling of the photocatalyst, and the ability to implement a flow rather than a “batch” process are some of the issues to consider for the successful implementation of a photocatalytic route-to-water decontamination. Large-scale wastewater treatment plants require a continuous operation unit able to process large volumes of liquid, which is generally difficult and economically disadvantageous with batch reactors. For these reasons, in this study, we tested the capabilities of solvothermal WO_3_ (WO_3_/st), prepared using a scalable solvothermal route and stabilized with a TiO_x_ adhesion layer, in the oxidative degradation of selected emerging chemical contaminants using both a batch method and a flow system based on a reactor packed with WO_3_/st-modified glass beads. 

## 2. Materials and Methods

### 2.1. Chemicals for Analytical and Photochemical Experiments

Drospirenone (DRO) was purchased from European Pharmacopoeia Reference Standard (purity 100%), 17-α-ethnylestradiol (EE2) from Dr. Ehrenstorfer GmbH (Augsburg, Germany) (purity > 96.3%), while oxazepam (OZ) was obtained from F.I.S. Fabbrica Italiana Sintetici S.p.a. (Vicenza, Italy) (purity 100%); Na_2_SO_4_ from VWR International S.r.l. (Milan, Italy) (purity > 99%), acetonitrile (ACN) and phosphoric acid were purchased from Merck KGaA (Darmstadt, Germany) (HPLC grade, purity > 99%), H_2_WO_4_ and oxalic acid dihydrate were purchased from Alfa Aesar (Haverhill, MA, USA) (purity 99.5–102.5%), hydrogen peroxide (purity 35%) and α-phenyl *N*-tert-butyl nitrone were purchased from Merck KGaA, lithium perchlorate was purchased from Acros Organics (Geel, Belgium) (purity 99%), hydrocloric acid was purchased from Carlo Erba Reagents S.r.l. (Milan, Italy) (37%). All the solutions were prepared using ultrapure waters (Milli-Q^®^ systems, Merck KGaA, Darmstadt, Germany).

### 2.2. Synthesis of WO_3_/st Films and Powder by Solvothermal Method

WO_3_/st nanocrystalline film was grown either on commercial glass spheres (GS), with a diameter of 4 mm, microscope glass slides (MGSs) or a fluorinated tin oxide glass (FTO glass, TEC 7 Ω supported on soda lime glass; Pilkington, Lathom, UK) substrate. All substrates were cleaned by ultrasonication for 10 min in 2-propanol prior to use. The preparation of the photocatalytic substrates was accomplished according to three sequential steps. 

Step 1: Before the deposition of WO_3_/st, the formation of a TiO_x_ nanometric adhesion layer (<5 nm) was accomplished by the room temperature hydrolysis of TiCl_4_ on the oxide supports (GS, MGS, FTO). Specifically, the soaking of the supporting surfaces in 0.4 M TiCl_4_ was carried out for 12 h for GS and MGS and 6 h in the case of FTO. After abundant rinsing of the glass substrates with deionized water, firing for 30 min at 450 °C in air afforded the desired TiO_x_ adhesion layer which, as we will demonstrate, does not block the collection of the photocarriers generated within WO_3_ upon band gap excitation (Figure S1). WO_3_ substrates obtained on top of the TiO_x_ adhesion layer are named WO_3_-Ti-substrate (i.e., WO_3_-Ti-FTO) and, unless otherwise indicated, all photocatalytic materials herein investigated are of the type WO_3_-Ti. 

Step 2: In the case of planar substrates, prior to solvothermal deposition, a WO_3_ seed layer with a thickness on the order of a few nanometers (typically 10–30 nm) was obtained by spin coating (9 s at 1000 rpm, 20 s at 2000 rpm); the precursor used for this process can be either 0.5 M peroxotungstic acid (PTA) or a colloidal precursor based on previously reported methods [29]. Such a precursor layer is converted into WO_3_ by firing at 550 °C in air. The best results were obtained with the peroxotungstic acid precursor. While the presence of the WO_3_ seed layer emphasizes the surface coverage and density of the photoactive film, its absence does not preclude the subsequent hydrothermal growth of the porous WO_3_/st layer. 

Step 3: The solvothermal (st) deposition of WO_3_ on top of the glass substrates was treated according to steps 1 and 2 (optional), followed a procedure described by Grimes et al. [35]. In a typical preparation of the synthesis of a dense WO_3_ photocatalytic layer based on oriented lamellar arrays or nanoflakes, the precursor solution was prepared by dissolving 1.25 g of H_2_WO_4_ in 10 mL of ultrapure water and 10 mL of 35% *w*/*w* H_2_O_2_ while heating and stirring bain–marie at 95 °C. In total, 18 mL of this H_2_WO_4_ solution (0.25 M), plus 1.2 g of oxalic acid and 3 mL of HCl (6 M), were mixed into 60 mL of acetonitrile and 15 mL of ultrapure water. Glass substrates, either flat slides or beads, were immersed in such a solution and heated at 180 °C in a Teflon-lined steel autoclave for a duration variable from 1 h to 30 min for glass spheres and 2 h for FTO. Besides the coated materials, WO_3_/st powder was also recovered from the bottom of the Teflon reactor, washed with distilled water, and dried in an oven at 60 °C for a few hours. All WO_3_/st-coated substrates were fired at 500 °C in air for 2 h to obtain the sintering and crystallization of WO_3_/st. Similar thermal treatment was used with the material in the form of a powder. 

### 2.3. Structural Characterization Techniques

#### 2.3.1. X-ray Powder Diffraction

Data from samples grown on flat surfaces were collected at room-temperature conditions (RT). Data collection was performed on a diffractometer (D8 Advance Davinci, Bruker Corporation, Billerica, MA, USA), working in Bragg–Brentano geometry, and equipped with a Cu-anode X-ray tube, Ni-filter to suppress the Cu Kβ component, and a LynxEye XE silicon strip detector (angular range of the detector window size = 2.585° 2θ) set to discriminate Cu Kα1,2 radiation.

Samples were loaded in a poly-(methyl methacrylate) specimen holder characterized by a cavity with adjustable depth to properly fit the microscope slide thickness, and scanned in a continuous mode from 5 to 90° 2θ, with step size of 0.02° 2θ and a counting time of 3 s per step. A knife perpendicular to the sample holder placed at a sub-millimetric distance from the sample was used to reduce the air-induced scattering.

Rietveld refinement. Collected XRPD patterns were modelled by means of the fundamental-parameter Rietveld approach (TOPAS v.5.0, Bruker Corporation, Billerica, MA, USA). Identified phases were modelled by carrying out a multiphase refinement in which only the scale factors and unit-cell parameters were varied. Instrumental parameters (e.g., goniometer radius, slit sizes, geometrical parameters of the X-ray tube, etc.) were used to calculate the instrumental contribution to the peak profiles, and specimen-related crystallite size information for each phase was extracted from the observed profile. An instrumental zero-error was fixed at the value, determined using the Si Standard Reference Material (640e, National Institute of Standards and Technology, Gaithersburg, MD, USA) standard, and the refinement included a specimen displacement correction and a Chebyshev polynomial to model the background.

#### 2.3.2. Scanning Electron Microscopy

The morphological characterization of WO_3_ films, on FTO substrate, was performed using a scanning electron microscopy (SEM, JSM-7001F FEG-SEM, JOEL Ltd., Tokyo, Japan) equipped with an energy-dispersive X-ray spectroscopy detector (EDXs, Oxford INCE PentaFETx3, Oxford Instruments Plc., Abingdon, UK). A 90° stub was used to define the thickness and the different layers of the electrode.

#### 2.3.3. Photoelectrochemical Characterization

Photoelectrochemical studies were carried out by exploiting the WO_3_ films grown on FTO. The typical photoelectrochemical cell was composed of an FTO/WO_3_ working electrode, a platinum wire counter electrode, and a saturated calomel electrode (SCE) reference electrode. Current/potential (J/V) curves were collected under simulated solar light using a solar simulator (ABET Technologies Inc., Milford, CT, USA) equipped with a AM1.5G filter, with a radiant power of 100 mW/cm^2^ and a 380 nm UV cut-off filter. The comparative measurements of WO_3_/st and WO_3_/coll were performed in an aqueous solution containing 0.7 M Na_2_SO_4_ as electrolyte, in a potential range between −0.3 V to 1.5 V versus SCE with a scan rate of 0.02 V/s. To reveal the effect of hole scavenging at the WO_3_/st interface by the selected contaminants, J/Vs were performed in an acetonitrile (ACN) solution, using 0.1 M LiClO_4_ as electrolyte, to bypass the low solubility of our target molecules in an aqueous solution. In this case, J/V curves were performed in a potential range between 0.1 and 1.7 V versus SCE with a scan rate of 0.02 V/s. J/V curves under pulsed illumination were obtained with an automatic shutter (SHB1T, Thorlabs, Newton, NJ, USA) at 0.3 Hz.

Cyclic voltammetry was performed in an aqueous solution containing 0.1 M LiClO_4_ and 0.01 M K_4_FeCN_6_. 

The incident photon-to-current conversion efficiency (IPCE) describes the photocurrent collected per incident photon flux as a function of the illumination wavelength. IPCE data, sampled at 10 nm intervals in the 310–500 nm range, were acquired in 0.7 M Na_2_SO_4_ using the same three-electrode configuration described above. Limiting photocurrents were collected at 1 V and 1.5 V for WO_3_/st and WO_3_/coll, respectively. Here, WO_3_/coll denotes a mesoporous WO_3_ electrode composed of nanoparticles (Figure S2), described in various previous publications [21,23].

The experimental setup used for photo-action spectra consisted of a monochromator (Applied Photophysics Ltd., Leatherhead, UK) equipped with a 175W Xe lamp (LuxteL-Sunoptic Technologies Inc., Jacksonville, FL, USA), various optical elements, a digital multimeter (34401A 6-1/2, Agilent Techologies Inc., Santa Clara, CA, USA), a potentiostat (552, AMEL S.r.l., Milan, Italy), and a calibrated silicon photodiode (OSD 7Q, Centronic Ltd., New Addington, UK).

Absorbed photon-to-current efficiency (APCE) spectra were computed, normalizing the IPCE value for the absorbed photons, after subtracting the reflected or transmitted photons.

A(λ) = 100 − R%(λ) − T%(λ)(1)

APCE is thus dependent on the quantum efficiencies of charge transport and interfacial separation defined as follows:

APCE(λ) = IPCE(λ)/a(λ) = η_transport_ η_interface_ (2)

considering that the efficiency of charge generation (ηe/h) is unitary in a semiconductor upon band gap excitation. 

#### 2.3.4. EPR 

EPR spin-trapping experiments were carried out with a Bruker MRD spectrometer equipped with a TE 201 resonator (microwave frequency of 9.4 GHz). The samples were suspensions of WO_3_ used for film fabrication in aqueous solutions containing α-phenyl *N*-tert-butyl nitrone (pbn, 5 × 10^−2^ M) as a spin trap. The samples were put into a flat quartz cell and directly irradiated (λ > 400 nm) in the EPR cavity with a medium-pressure mercury lamp. No EPR signals were obtained in the dark and during the irradiation of the solution in the absence of WO_3_. 

### 2.4. Photochemical Degradation Experiments

Batch experiments. Photodegradation tests were performed using the batch method, where a solution with a known concentration of a contaminant is placed in contact with a known quantity of WO_3_/st powder (70 mg) as a photocatalytic dispersion. The photodegradation tests were carried out in 20 mL flasks under constant stirring for 300 min at room temperature (Figure S3). The solutions were irradiated using a solar simulator (ABET Technologies Inc., Milford, CT, USA, equipped with a AM1.5G filter, with a radiant power of 100 mW/cm^2^ and 380 nm UV cut-off filter), a device that provides illumination approximating natural sunlight. For each solution containing 4 µg/mL of a drug (EE2, DRO and OZ) in a 0.7 mM Na_2_SO_4_ aqueous solution at pH = 6 ± 0.30, the following four tests were carried out under different conditions: (i) a dark test to evaluate the adsorption of contaminants at the glassware walls; (ii) a dark test, in the presence of powdered WO_3_/st, to evaluate the adsorption of drugs at the surface of the solid photocatalyst; (iii) an irradiation test without WO_3_/st to verify the direct photochemical degradation of each contaminant; and (iv) irradiation cycles in the presence of the photocatalyst to reveal the photodegradation deriving from the WO_3_/st activity. At the end of each experiment, the separation of the supernatant was achieved by centrifugation, and the supernatants containing OZ and DRO were analyzed by HPLC-DAD (see Section 2.6), whereas EE2 solutions were analyzed by HPLC-FLD (see Section 2.7). 

**Figure 2:**
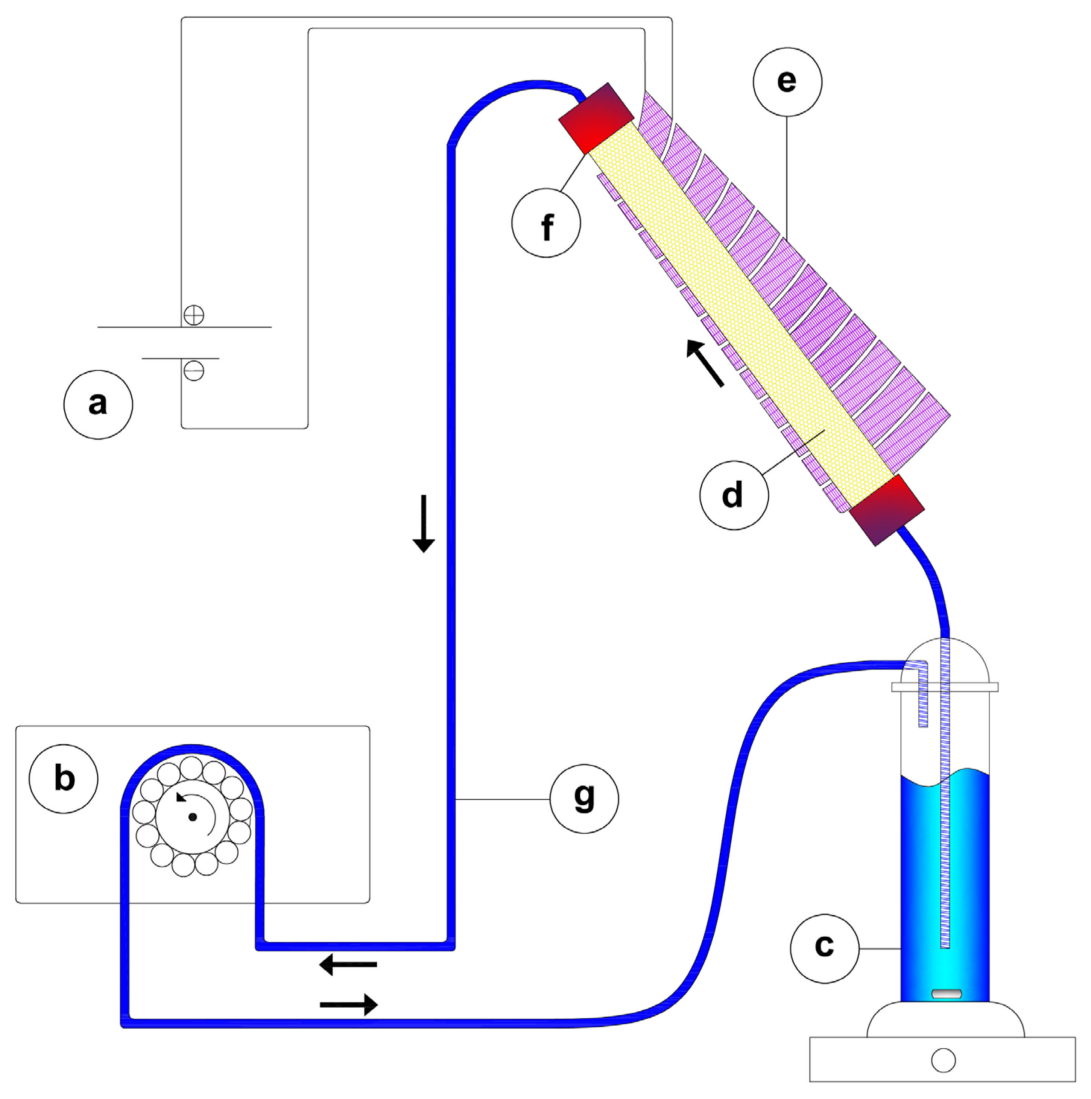
Flow system: (a) 24 V direct-current generator (DC); (b) peristaltic tube pump with two sprung rollers; flow rate set at 7 mL min^−1^; (c) mixing system on magnetic stirrer; (d) Ø3 mm glass spheres, WO_3-_coated (WO_3_/st-GS), confined in a glass column with internal Ø19 mm and outer Ø 22 mm; (e) 24 V LED source, λ = 395–400 nm, completely enveloped column; (f) PE screw cap to fix a frit in PP with 2 mm grill in order to lock spheres; and (g) tubing, internal Ø4 mm and outer Ø 7 mm.

Flow system experiments. Moving from batch experiments to the flow system, a 10-fold scale-up in terms of volume was achieved, reaching a total treated water volume of 200 mL. The apparatus was composed of a glass flask, containing the polluted solution, a magnetic stirrer and a glass column containing glass spheres (GSs) coated with WO_3_/st (WO_3_/st-GS) (Figure 2). The column was completely enveloped by an LED stripe (395 < λ < 400 nm, Figure 2e) to obtain a uniform illumination of the whole packed tube and increase the probability of photon absorption by the photocatalyst along the whole tube length. The emission range of LEDs was selected in order to maximize the efficiency of photoconversion, peaking, in the WO_3_ photocatalyst, at the edge of the visible spectrum (Figure 2d). The average irradiance generated by the LED strip was 49 mW/cm^2^. 

The water flow was controlled by a peristaltic pump to 7 mL/min. Typical illumination cycles of 480 min under electrolyte recirculation conditions at room temperature were carried out by using an aqueous test solution containing 4 µg/mL of the selected drugs in 0.7 mM Na_2_SO_4_ at pH = 6.00 ± 0.30 (Five Easy F20, Mettler-Toledo International Inc., Greifensee, Switzerland). Similar to batch tests, various control experiments under different conditions were performed. The flow system connection tubes were selected in consideration of a low surface affinity towards the various contaminants in order to minimize drug losses by adsorption. To keep a tight packing of WO_3_/st-GS in the column, PE screw caps and PP frits were placed at the inlet and outlet of the column. The electrolyte was sampled during the degradation experiments to evaluate the kinetics of the process by verifying the concentration of drugs in the solution and the formation of by-products.

### 2.5. Kinetic Models

Chemical kinetics are a part of science with the theoretical purpose of determining the mechanism of a reaction. Among the different models to study and understand the mechanism, the experimental data were well described by the first order kinetic model (3). First order reactions are those reactions whose rate depends on the concentration of a single reactant raised to an exponent of 1. The mathematical equation is as follows:

ln[C]/[C_0_] = −kt (3)

where, [C] is the reagent concentration at time t, [C_0_] is the initial concentration of the reagent, k is the kinetic constant and t is the time of reaction [36,37].

### 2.6. HPLC-DAD Analysis

OZ and DRO removal was evaluated by HPLC-DAD analysis. The apparatus was composed of two pumps (515 HPLC pump, Waters Corp., Milford, MA, USA), Rheodyne injectors (IDEX Corporation, Northbrook, IL, USA) with 20 µm loop and a photodiode array detector (996 DAD, Waters corp., Milford, MA, USA). The column selected was C18 100A 150 × 4.6 mm with a particle size of 5 µm (Kinetex SuXB, Phenomenex, Torrance, CA, USA). The mobile phase was a mixture of acetonitrile (ACN) and ultrapure H_2_O, and the analyses were performed under isocratic conditions. The flow rate was 1 mL/min, and the injection volume was 20 µL. For the OZ molecule, the mobile phase mixture was 40% ACN and 60% H_2_O, while for the DRO molecule, the eluent was 50% ACN and 50% H_2_O. OZ and DRO were detected at 227 nm and 265 nm, respectively.

### 2.7. HPLC-FLD Analysis

EE2 analysis was performed using an HPLC (1290 Infinity, Agilent Technologies Inc., Santa Clara, CA, USA) equipped with a binary pump, autosampler, thermostatic column compartment, and fluorescence detector (FLD 1290 Infinity II, Agilent Technologies Inc., Santa Clara, CA, USA). The selected column was C18 100A 150 × 4.6 mm with a particle size of 5 µm (Kinetex SuXB, Phenomenex, Torrance, CA, USA). The column compartment temperature was set to 25 °C. The eluent mixture consisted of 70% ACN and 30% ultrapure water, and the analysis was performed under isocratic conditions; the flow rate was 1 mL/min, and the injection volume was 20 µL. The detection conditions were set to an excitation wavelength of 280 nm and an emission wavelength of 310 nm.

### 2.8. HPLC-MS Analysis

HPLC/MS analyses were carried out by means of Surveyor micro-HPLC hyphenated to a linear trap quadrupole mass spectrometer (LTQ XL, Thermo Fisher Scientific Inc., Waltham, MA, USA). The HPLC was composed of a solvent delivery system, a quaternary pump and an autosampler. The LTQ system was equipped with an electrospray ionization (ESI) ion source. The mobile phase was obtained by mixing ACN/0.1% *v*/*v* formic acid with ultrapure water/0.1% *v*/*v* formic acid according to the following gradient program: 0 min 10% ACN, 0–10 min 10–90% ACN, and 10–12 min 90% ACN. Then, the column was reconditioned at 10% ACN until reaching 15 min of elution time. The flow rate was 150 μL/min. The column was C-18 100 mm × 2.1 mm × 2.1 μm (Supelco, Bellefonte, PA, USA). The injection volume was 2 μL for all standards and samples. MS experimental conditions were as follows: spray voltage 5 kV, capillary temperature 275 °C, capillary voltage 28 V and tube lens 50 V for positive ESI conditions (OZ and DRO); spray voltage 5 kV, capillary temperature 275 °C, capillary voltage −50 V and tube lens −137 V for negative ESI conditions (EE2).

## 3. Results and Discussion

### 3.1. Morphological and Structural Properties of Nanostructured WO_3_

The geometrical constraints of the GS make it impossible to perform a reliable structural and morphological characterization of WO_3_ nanostructures decorating these curved supports. Nevertheless, the structural properties of WO_3_ films obtained on flat surfaces, consisting of both FTO and simple glass, are considered representative of the general properties of the WO_3_ coating on the glass beads. In addition, the use of conductive FTO allows us to quantify, via photoelectrochemical measurements, the performance of these WO_3_ substrates in generating reactive charge carriers when excited with suitable optical frequencies. 

#### 3.1.1. Electron Microscopy 

**Figure 3:**
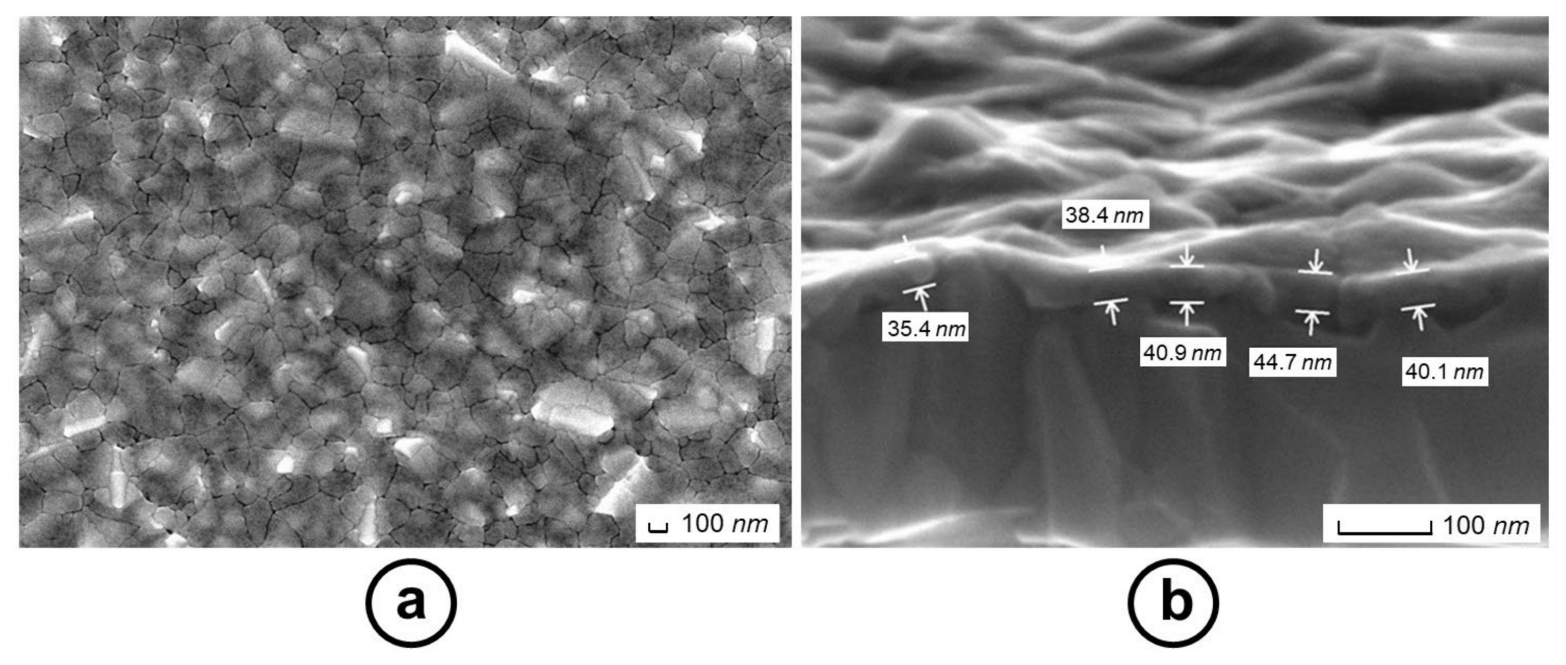
Morphology of the TiO_x_-treated WO_3_ seed layer on FTO support imaged from the top (**a**) and from the side (**b**), showing the thickness of the deposit.

The presence of the TiO_x_ adhesion layer is not clearly discernible on the FTO surface, indicating that its thickness is below the instrumental detection limit (5 nm in cross section mode on this type of surface) of our SEM. At such low thickness, the crystalline and band structure of TiO_x_ is probably unable to fully develop, resulting in a defective layer which allows electron percolation to the TCO collector to occur, as shown by cyclic voltammetry with a fast Fe (II)/III) redox probe (Figure S1). Instead, the compact WO_3_ seed layer can be clearly seen from Figure 3. The seed layer produced with the PTA precursor conformally covers the FTO substrate, which has an overall rough and irregular appearance due to the presence of large FTO crystals (hundreds of nanometers in size). The seed layer displays a thickness of about 30–40 nm, as shown in Figure 3b, and is quite homogeneous, despite the presence of cracks, in particular in the valleys between adjacent FTO crystallites, wherein the liquid precursor tends to accumulate, giving rise to a locally thicker WO_3_ film which fractures upon thermal sintering. 

The effect of the TiO_x_ adhesion layer is macroscopically evident from the mechanical properties of the photoactive films at the end of the solvothermal growth and annealing (Figure S4a,b). In the WO_3_/st-FTO (WO_3_/st = solvothermal WO_3_) samples (that is, steps 2 and 3 without the TiO_x_ adhesion layer), uncoated islands appear as a result of the thick WO_3_ film detaching from the TCO surface. Moderate magnification optical microscopy shows that the WO_3_ film crinkles and peels off the surface because of poor adhesion, or thermal stresses during the annealing, leaving exposed FTO underneath. Actually, the WO_3_-FTO shown in Figure S4b is one of the few samples where the solvothermal WO_3_ layer did not detach completely from the FTO ohmic contact upon rinsing and annealing. When the same procedure is carried out after the Ti(IV) treatment (step 1), affording the sample WO_3_/st-Ti-FTO, one obtains an electrode which remains solidly and uniformly coated after the rinsing/annealing cycle. The hydrophilic TiO_x_ layer considerably stabilizes the WO_3_ adhesion to the electrode, being characterized by the presence of a higher-than-FTO density of surface OH groups. Thus, multiple oxo bridges with WO_3_ could be formed, guaranteeing a robust anchor to the FTO of the latter. 

**Figure 4:**
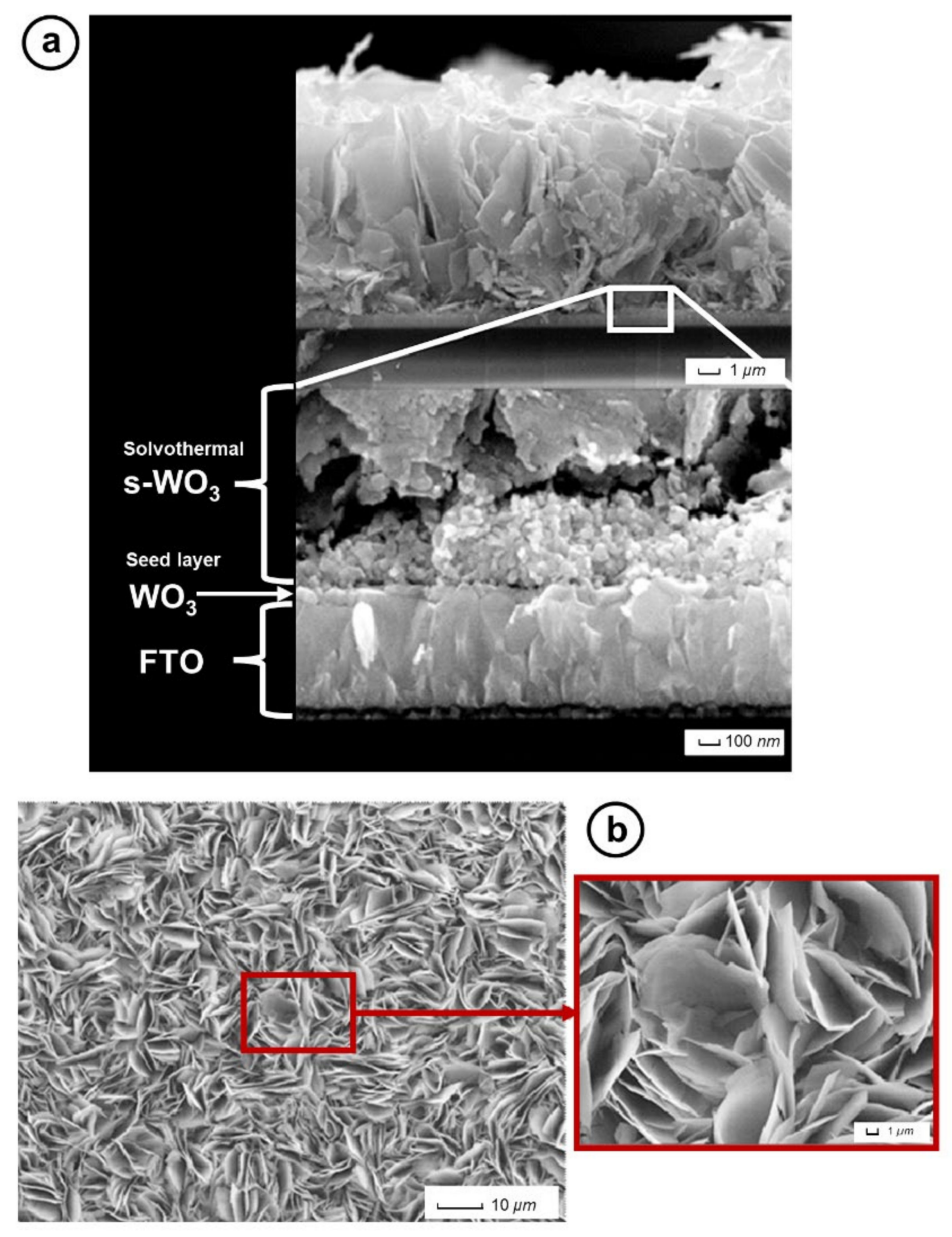
SEM imaging of WO_3_-TiO_x_-FTO electrode in cross sectional (**a**) and top views (**b**). The magnification of the top view allows us to appreciate the thickness of the WO_3_/st leaves, of less than 100 nm, compared to their length and width of several microns.

The typical surface of a WO_3_/st-Ti-FTO electrode is characterized by a dense coverage of WO_3_ leaves and lamellae, roughly vertically aligned with respect to the surface, and arranged in somewhat organized superstructures which may resemble flowers or flakes. These leaves are relatively thin (<100 nm) (Figure 4b) compared to their length and width, which extend for several microns (Figure 4a) and could, thus, be essentially considered 2D structures. The inspection of the cross section of the electrode reveals that these lamellae extend considerably in length, taking up most of the film thickness (about 8 μm) and confirm their close packing and roughly vertical alignment with respect to the FTO surface. A closer inspection of the cross section reveals a disordered layer, composed mostly of nanoparticles, with the overall thickness of few 100 s of nanometer between the roots of the leaves and the seed layer. This rubble-like layer could be formed either by the partial collapse of the lamellae, or by the precipitation of WO_3_ during the initial stage of the solvothermal growth before thermal equilibrium is reached. Nevertheless, it is clear that either in electrodes or decorated glass supports illuminated from the top (electrolyte side), photon absorption will occur mostly within the 2D structures, which will be the primary actors in the charge transfer dynamics involving WO_3_/st. In the absence of the TiO_x_ adhesion layer, the same lamellar morphology of the WO_3_ layer of Figure 4 is observed (Figure S5), although a nearly complete detachment of the WO_3_ film from the FTO ohmic support is observed. EDS analysis confirms the expected stoichiometry of WO_3_, with tungsten and oxygen as the only significant elements in the elemental composition of the nanostructured film, appearing in a 1:3 molar ratio (Figure S6). 

#### 3.1.2. X-ray Diffraction 

X-ray diffraction (XRD) data confirmed that WO_3_/st on the Ti-FTO substrate was composed by high-quality monoclinic WO_3_, with a P 21/n space group, formed during high-temperature annealing with cell parameters of a = 7.320 Å, b = 7.518 Å, c = 7.670 Å (V(Å3) = 422.1), β = 90.4° (WO_3_ database code ICSD 80056) and was marginally different from the data collected on other types of standard WO_3_ electrodes produced in our laboratory [37] (our colloidal standard (WO_3_/coll) affords a = 7.303 Å, b = 7.536 Å, c = 7.686 Å (V(Å3) = 422.9), β = 90.4° (WO_3_ database code ICSD 80056), and consistent with the results reported by Grimes [35] in his paper. This confirms that the TiO_x_ adhesion layer does not interfere with the subsequent solvothermal growth. Crystallite size is much smaller than the size of a whole lamella, being only 25.09 nm. Thus, every leaf is formed by several nanometer-sized WO_3_ crystallites. 

**Figure 5:**
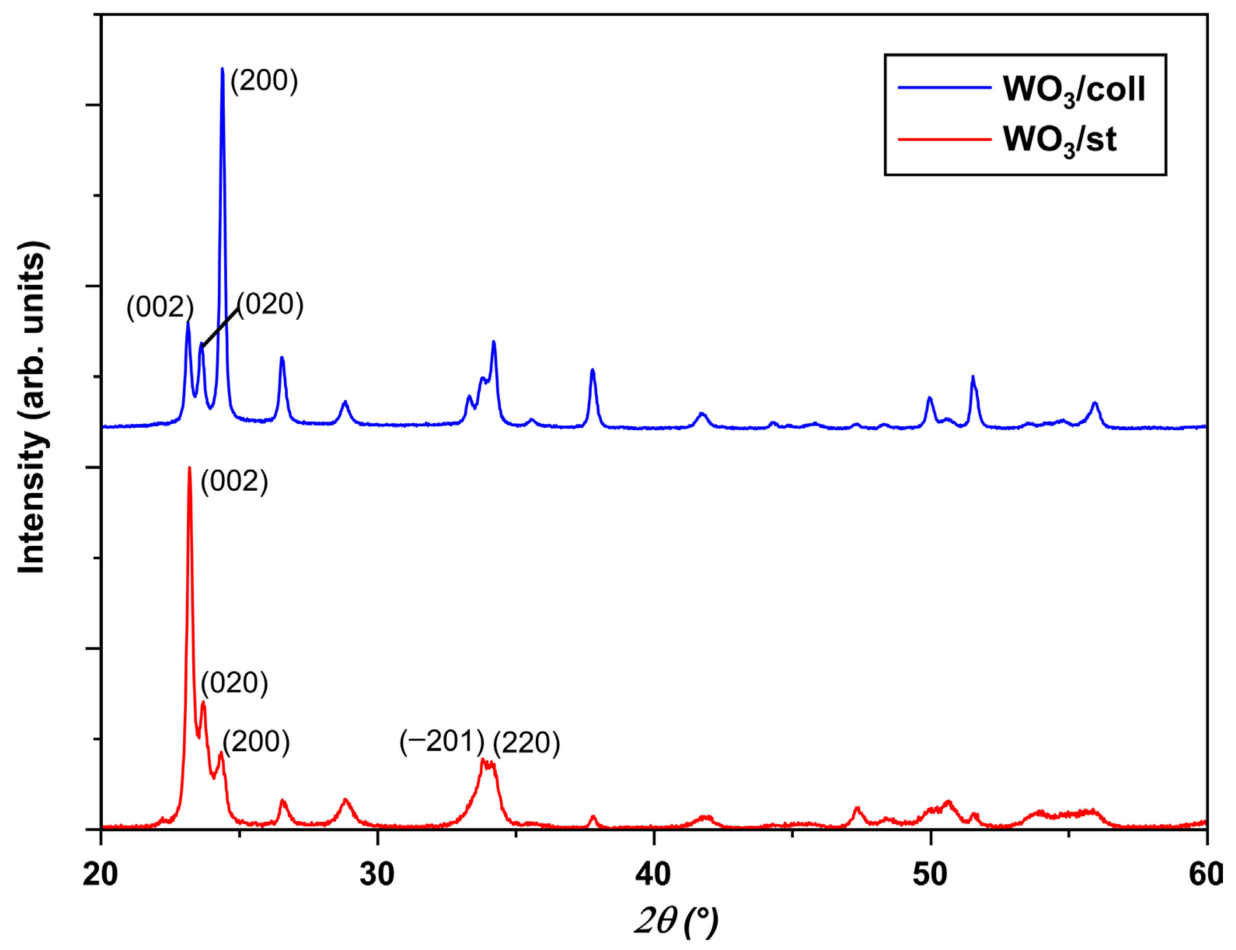
XRD patterns recorded on FTO substrate compared to the WO_3_/coll standard of known art produced in our laboratory.

The diffraction peak at 2θ of 23.2°, 23.6° and 24.4° corresponds to the (002), (020) and (200) facets (see Figure 5). The two samples show different preferentially exposed facets which are (200) for WO_3_/coll and (002) for WO_3_/st. A possible mechanism for solvothermal growth that explains the preferential (002) facet exposure in WO_3_/st is proposed by Gong et al. [33], who state that, during the solvothermal reaction, the Cl^−^ functions as a kind of capping ion analogous to what was reported for F^−^ [38,39,40]. Since the (002) facets have a larger surface energy than (020) and (200) ((002) (1.56 Jm^−2^) > (020) (1.54 Jm^−2^) > (002) (1.43 Jm^−2^)), Cl^−^ is preferably adsorbed on the (002) facets. This adsorption weakens the surface affinity for the precursor to this orientation, thus inhibiting the crystal growth rate along the perpendicular to these planes, resulting in the increased exposure of the (002) facets. It is well known that the reactivity of a photocatalyst is influenced by its surface atomic and electronic structure [40], so the preferential exposure of (002) instead of (200) family of planes could strongly influence the photocatalytic performance of these WO_3_ structures. Indeed, as reported by Gong et al. [33], the preferential exposure of (002) facets was found to improve the photoelectrochemical performance of WO_3_ photoanodes. According to Wang et al. [34], the preferential exposure of the highly active (001) facets that belong to the same {001} family of planes as (002) facets endows WO_3_ nanocrystals with significantly enhanced photocatalytic activity, which can be attributed to the stabilization of reactive oxygen species and the reduced recombination of photogenerated electrons and holes at these interfaces. 

As concerns the WO_3_/st-Ti grown on simple glass slides, a comparison of the collected X-ray diffraction patterns at RT is reported in Figure 5. 

A qualitative phase analysis of the collected patterns was performed by means of the AXS EVA software (v.6.0.0.7) (Bruker Corporation, Billerica, MA, USA). Both samples were biphasic and composed of tungsten oxide WO_3_ in two polymorph modifications, namely monoclinic (s.g. P2_1_/n), which is largely prevalent (>95%) and hexagonal (s.g. P6_3_/mcm) (<4%). The hexagonal phase was observed previously in materials obtained through other types of hydrothermal processes, and was found to be active in oxygen evolution processes as well [41]. The presence of hexagonal/monoclinic junctions was considered beneficial for improved charge separation by some authors [42]. Prolonged exposure of WO_3_/st to pH 7 water (WO_3_-washed) does not change the compositional profile substantially, indicating that both phases persist on the surface of glass supports during the employment of the photocatalyst in neutral aqueous media. The Rietveld refinement plots for both samples are shown in Figures S7 and S8. Quantitative phase analysis and unit-cell parameters, as well as the crystallite size of the main phases, are summarized in Table 1.

The result of the XRD investigation allows us to conclude that the nature of the support (either simple glass or TCO) does not affect the crystalline nature of WO_3_/st by a large margin. Coherent scattering domains are also similar (ca. 20 nm) with either SnO_2_:F or borosilicate glass supports, with different surface chemistry. This is probably due to the fact that both surfaces are modified, to stabilize the adhesion of WO_3_/st, with the same adhesion (TiO_x_) and seed (WO_3_) layers. A largely prevalent monoclinic phase is found with both substrates, which is expected to play a major role in the photoinduced processes activated upon the optical excitation of the semiconductor. Furthermore, the solvothermal coating method is surprisingly easy to extend to the functionalization of a variety of substrates of shapes other than planar (i.e., irregular porous supports, glass beads) (Figure S9). 

### 3.2. Photoelectrochemical Properties 

Evaluating the photoelectrochemical response of WO_3_/st (Figures S10 and S11) will allow us to judge the ability of these WO_3_ films to achieve efficient electron/hole separation, which is the primary goal and crucial for any solar energy conversion device, irrespective of its intended use in either solar fuel generation or environmental remediation processes. In addition, the information gained from photoelectrochemical experiments can be extended to the open circuit (i.e., photocatalytic) case where the efficiency figures are more elusive to obtain without ambiguity. The primary efficiency figure for this type of photoelectrochemical application is the magnitude of the current density, which is dependent on the rate of hole injection into the electrolyte, yielding OH radicals as the consequence of water oxidation. The generation of ·OH radicals in WO_3_ aqueous suspensions under visible light illumination (λ > 420 nm) was confirmed by EPR spin-trapping measurements. Few seconds of photoirradiation of the WO_3_ suspension in the presence of pbn causes the prompt formation of a triplet of doublets characterized by coupling constants aN = 15.4 G, aH = 2.7 G, which is shown in Figure S12. The coupling constant values are in agreement with the trapping of ·OH by pbn to form the paramagnetic adduct [pbn-OH]. These results confirm that photoexcited WO_3_ is able to initiate water oxidation by producing OH radicals. First, we can observe that the WO_3_/st-type electrode reproducibly yields (max. relative standard deviation ca. 10%) much better results with respect to the state of the art [35] from which we adapted our preparative procedure, with an average limiting photocurrent of ca. 2.2 mA/cm^2^. This corresponds to a ca. 60% improvement over the reported case. WO_3_/st is also a better performer compared to one of our best standard WO_3_/coll (synthesis and characterization was previously published) [21] by a factor of ca. 40% (Figure S11). Interestingly, the WO_3_/st J/V curve is generally steeper compared to the standards, suggesting low charge transport resistance across the oriented electrode and good interfacial charge separation efficiency. The global resistance R = (dV/dJ) figure of merit, comprising both the interfacial and the charge transport resistance, is indeed lower by a factor of 2 in the WO_3_/st material (Nyquist Plots were compared in Figure S10). This confirms that the TiO_x_ adhesion layer offers a stable anchor for prolonged photoelectrode operation while being sufficiently permeable to electrons to allow for their efficient collection. 

**Figure 6:**
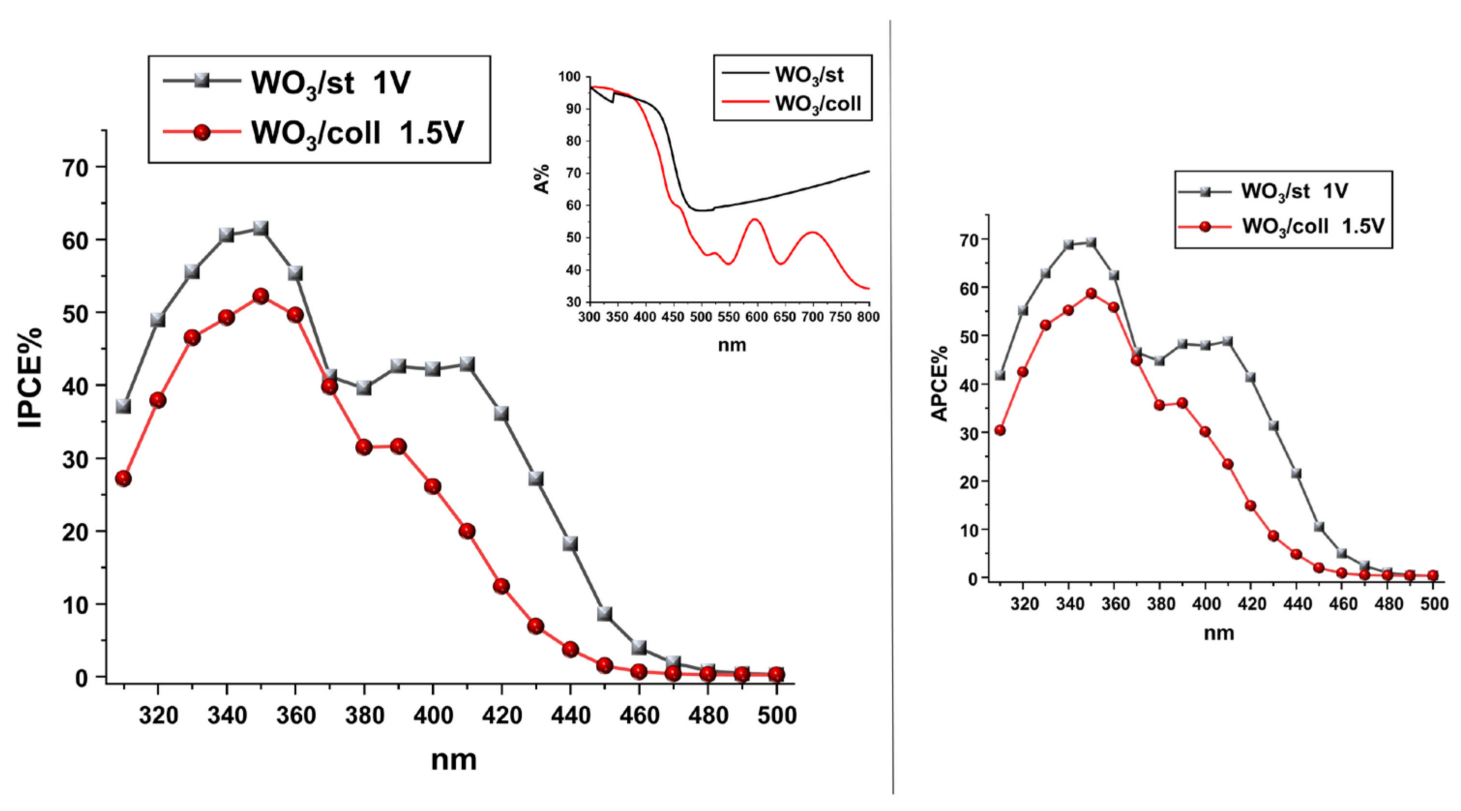
IPCE% and APCE% of WO_3_/st and WO_3_/coll under, respectively, 1 V and 1.5 V bias in 0.7 M Na_2_SO_4_. Their absorptance spectrum (A = 100 − R% − T%) is shown in the inset.

Current potential results were complemented by photon-to-electron conversion spectra: IPCE and APCE (Figure 6). IPCE% of WO_3_/st and WO_3_/coll are compared under 1 V and 1.5 V applied potential. We note that the limiting value of WO_3_/st is reached with a reduced overpotential of ca. 0.5 V compared to WO_3_/coll, consistent with the lower charge transfer resistance. First, we observe that IPCE is in good agreement with the J/V results, showing that the area subtended by the WO_3_/st photoconversion is ca. 30% higher than that of WO_3_/coll. It is also interesting to note that most of the improvement of WO_3_/st comes from a better conversion in the visible region, from 400 to 500 nm. APCE% spectra confirm, for the WO_3_/st, intrinsically higher charge transfer and charge transport efficiencies with respect to WO_3_/coll. For the former, we record APCE≈ 70% in the near UV, meaning that 70% of the photons absorbed in such a region is converted into useful charge carriers for driving an energetically demanding redox reaction. In the blue visible region, such efficiency is still significant, with a maximum value of the order of 50%. Consistent with the gap of ca. 2.7 eV, photoconversion ceases completely around 500 nm. The energy gap estimated from the photoaction spectra was confirmed by Tauc analysis (Supplementary Materials, Figure S13), displaying a reasonably linear region and affording an indirect band gap of 2.63 eV and a direct gap of 2.88 eV. 

We probed the impact of the presence of DRO, EE2 and OZ on the J/V characteristic of WO_3_ to gain direct indication of the photoelectrochemical degradation of these molecular targets. We note that the low solubility of DRO, EE2 and OZ in aqueous solution makes the direct observation of hole scavenging by these species impossible to be clearly observed by simply recording the photocurrent. For this reason, we explored the photoelectrochemical response of WO_3_ in ACN/0.1 M LiClO_4_, where concentrations of DRO, EE2 and OZ could be raised up to 0.01 M. The photocurrent recorded with blank ACN/LiClO_4_ solutions is due to both the oxidation of the residual water present in non-anhydrous ACN and the direct oxidation of the organic electrolyte [43].

**Figure 7:**
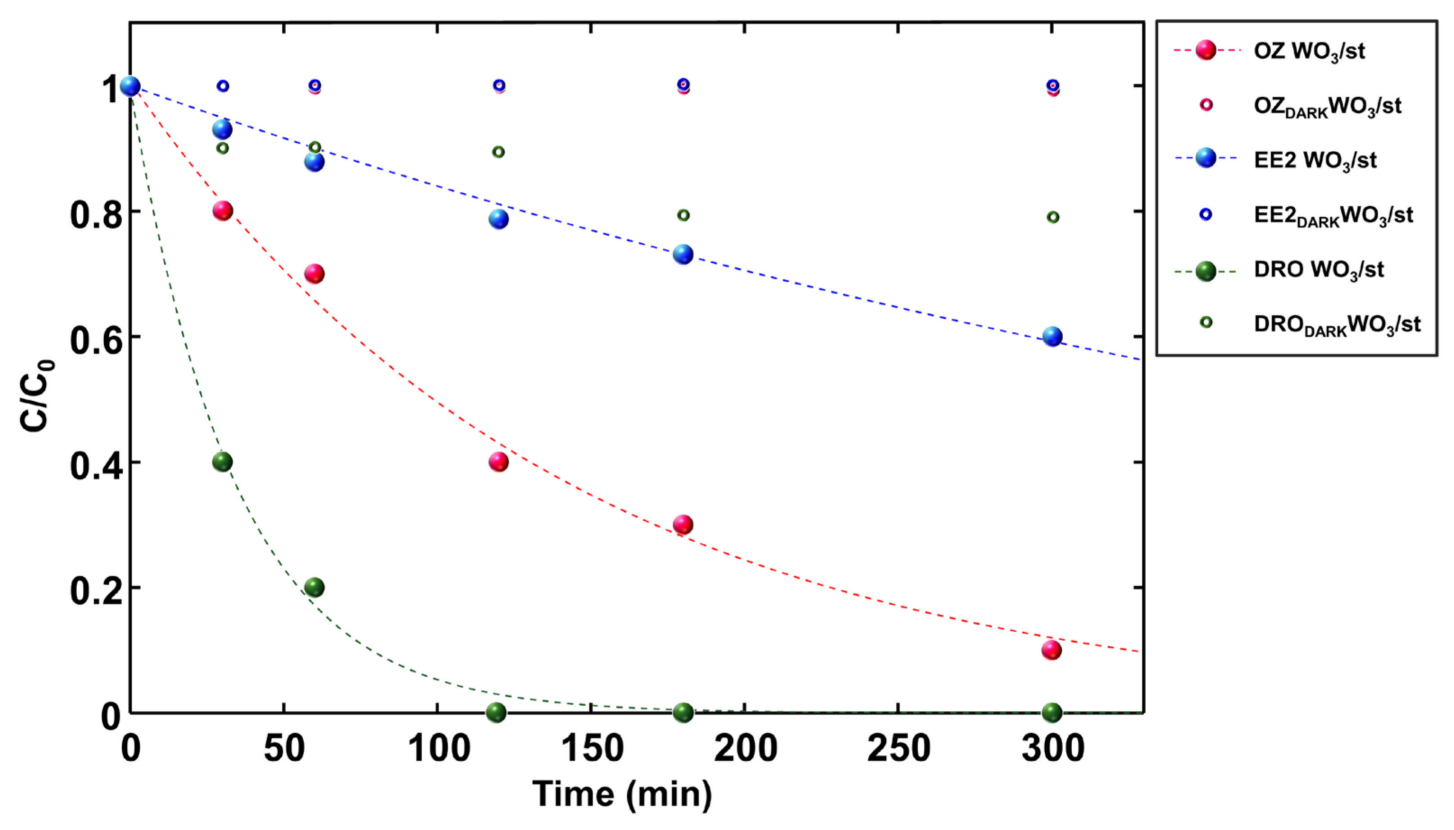
Photodegradation kinetics in batch system, data expressed as C/C_0_ vs. time; EE2 WO_3_/st, DRO WO_3_/st, OZ WO_3_/st represent the data of the irradiation experiments, EE2_DARK_WO_3_/st, DRO_DARK_WO_3_/st, OZ_DARK_WO_3_/st represent the data in dark conditions. Each solution was prepared at an initial concentration of 4 mg L^−1^.

In Figure S14, it is possible to observe, that, when DRO EE2 and OZ are present in the electrolyte, a 300 mV cathodic shift of the photoanodic onset potential occurs, associated with an increased photocurrent in the 0.2–0.8 V vs. SCE interval, a region of the J/V curve which is usually affected by electron/hole recombination losses. Both these effects are consistent with the fast capture of either holes or photogenerated radicals by the selected target organic species, resulting in their irreversible oxidation. We also point out that no dark electrochemical processes resulting from the electrochemical oxidation of DRO, EE2 and OZ on the FTO/WO_3_ surface are observed up to 1.8 V vs. SCE. We have thus provided direct evidence of the occurrence of the photoelectrochemical oxidation of DRO, EE2 and OZ at the illuminated WO_3_ interface. Moreover, in the presence of DRO, EE2 and OZ, we generally observe a drop in the limiting photocurrent of the photoanodes, a fact that we have tentatively explained with the adsorption of the drug oxidation intermediates on the WO_3_ surface, leading to a decreased active area of the photoelectrodes. In addition, EPR spin-trapping experiments were carried out on a WO_3_ aqueous suspension containing DRO irradiated for few seconds in the presence of DMPO (see Section 2.3.4). It was observed the formation of the quartet (1:2:2:1, aN = aH = 14.8 G) was characteristic of the trapping of OH• radicals by DMPO (see Figure 7). After a few minutes, a triplet of doublets (aN = 15.36 G, aH = 20.28 G) appeared; these signals are possibly due to an adduct of a carbon radical originated from DRO and DMPO. 

### 3.3. Photochemical Degradation of DRO, EE2 and OZ

Batch experiments. To study the photocatalytic removal of the selected pharmaceuticals, batch experiments with and without the WO_3_/st photocatalyst, under irradiation and in dark conditions, were performed under the conditions described in Section 2.4. The concentration of the three tested drugs was determined by HPLC, as reported in Section 2.4, Section 2.6 and Section 2.7. The determined concentration (C)was divided by the initial concentration (C/C_0_). In the case of EE2 and OZ, in the absence of illumination, we demonstrated that the photocatalytic system does not contribute to drug adsorption. Indeed, the concentrations of neither EE2 nor OZ vary significantly under dark conditions, regardless of whether the catalyst is present or absent, and the ratio C/C_0_ is close to 1. A reduction of approximately 20% (C/C_0_ roughly 0.8) was observed only for the DRO molecule (as shown in Figure S15), and was thus likely attributable to an effective adsorption event. When comparing the results from the tests conducted in the darkness, it became evident that this specific pollutant is adsorbed onto the photocatalyst. Indeed, the data from dark conditions (in the absence of WO_3_/st) revealed no alterations in the DRO concentration, whereas a significant change was noted upon the addition of the photocatalyst (Figure S15). Moreover, the outcomes of irradiation tests conducted without the WO_3_/st powder indicate that the direct photochemical degradation of pollutants is in no case significant. On the contrary, a remarkable decrease in the concentration of drugs was observed only when the test solution was irradiated in the presence of WO_3_/st. Figure 7 shows the degradation kinetics of the three drugs, clearly indicating the occurrence of a photocatalytic process in the presence of the WO_3_/st suspension.

Flow system experiments. For each pollutant (DRO, EE2 and OZ), the contribution to adsorption was investigated by evaluating the differences in the concentration determined by the HPLC of the contaminants in solution before and after contact with the glass spheres coated with WO_3_/st (WO_3_/st-GS). In these experiments, 20 g of photoactive spheres were placed in a vial and kept in contact with 75 mL of aqueous solution for 420 min in the dark. The experimental data obtained showed that, for DRO, the uptake onto the WO_3_/st-GS in (Figure S15) is negligible. Possibly, the difference in the adsorption of DRO onto the supported photocatalyst system WO_3_/st-GS is even lower than that onto the WO_3_/st due to the higher surface area of the latter one. 

**Figure 8:**
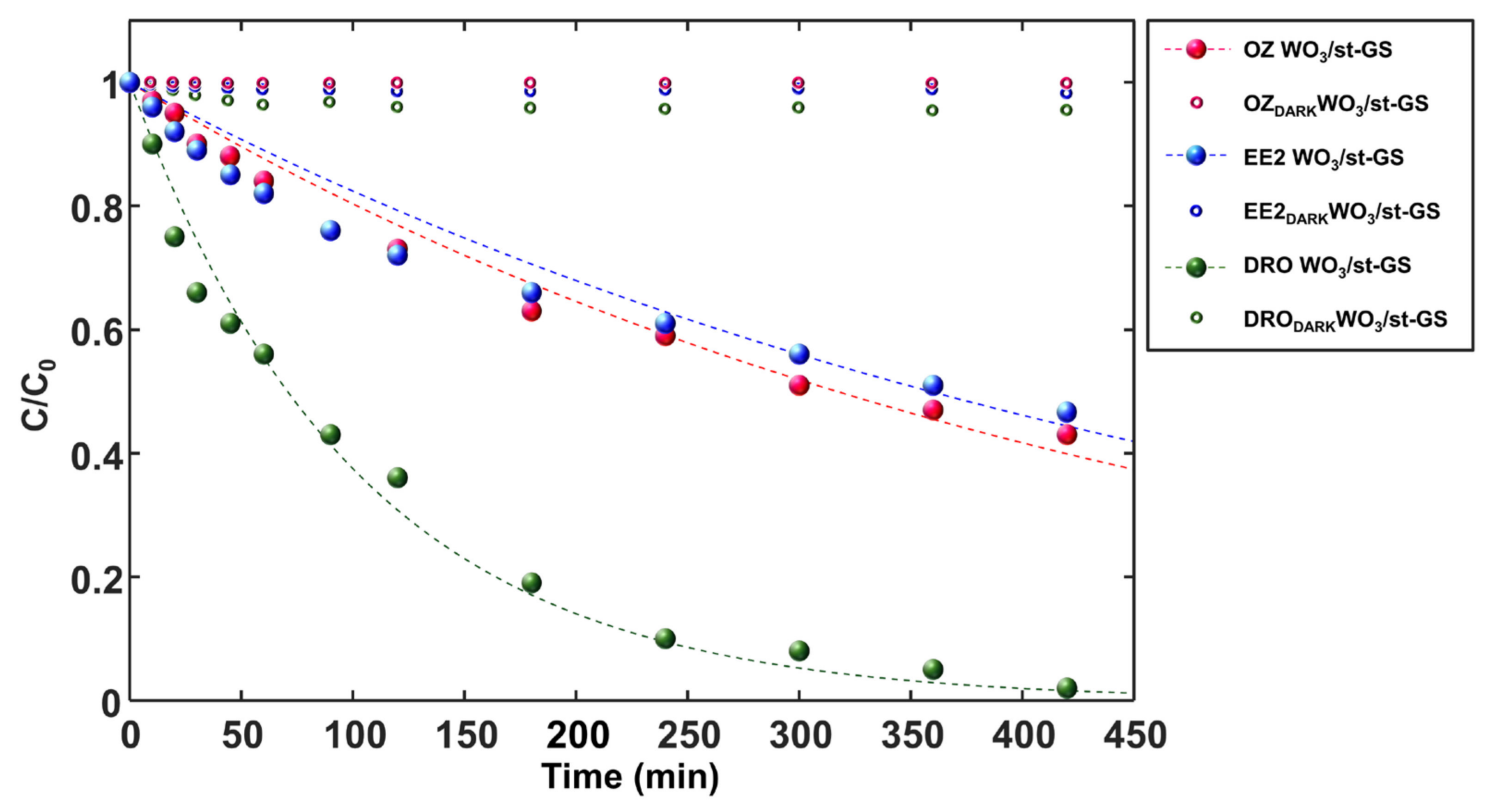
Drug photodegradation kinetics in the flow reactor, data expressed as C/C_0_ vs. time; EE2 WO_3_/st, DRO WO_3_/st, OZ WO_3_/st represent the data of the irradiation experiments, EE2_DARK_ WO_3_/st, DRO_DARK_WO_3_/st, OZ_DARK_WO_3_/st represent the data in dark conditions. Each solution was prepared at an initial concentration of 4 mg L^−1^.

Experiments on the degradation kinetics by using the flow system (see Figure 3) were carried out by using 200 mL of 0.7 mM Na_2_SO_4_, aqueous solution containing 4 mg L^−1^ of a drug to simulate the surface water conditions for what concerns both salinity and pH (see Section 2.4) [36]. Concerning the light absorption efficiency in the flow photoreactor, we have estimated, by exploiting diffuse reflectance measurements, that an attenuation of 400 nm light of ca. 60% (Figure S16) due to absorption by WO_3_ is achieved within an optical path of 1 cm. This is a similar attenuation to that observed with the WO3st suspension in static experiments (Figure S17). Considering that the diameter of the photoreactor is 2.2 cm, we obtain, along the radial coordinate, a nearly complete absorption (ca. 96%) of 400 nm light within the active volume of the reactor. In Figure 8, the results obtained using the flow system under irradiation are shown. Overall, a substantial drop in the concentrations of all pharmaceutical targets is observed upon the treatment of the test solutions in the flow reactor. However, the DRO and OZ degradation rates were smaller than those found in batch experiments. Possibly, the slower kinetics are due to the shorter contact time between the solution and the WO_3_/st photocatalyst in the flow system with respect to the batch experiments. It has already been reported that, in a flow reactor, the immobilization of WO_3_ on a substrate leads to a lower activity [44]. Experimental data were fitted using a pseudo-first order (PFO) (1) kinetic equation. In the literature, many cases of pseudo-first order photocatalytic decomposition of organic compounds at similar concentrations in a flow reactor are reported [45,46]. In Table 2, the results obtained from both batch and flow experiments are shown. 

From the data in Table 2, it can be seen that a WO_3_-based photocatalyst is able to degrade all the selected drugs. Indeed, a WO_3_-based photocatalyst, much like other semiconductor catalysts, is not selective, since the degradation reaction occurs through reactions with hydroxyl radicals (OH•), which are a strong oxidant characterized by an oxidation potential equal to E° = +2.1 V vs. NHE. In the case of EE2, the kinetic constants estimated from the flow and batch experimental data are higher than those of DRO and OZ. This could be explained by the presence, in the EE2 structure, of reactive OH groups and electron-rich carbon atoms. These functional groups likely serve as primary sites for initial reactions in the advanced oxidation process, potentially necessitating a shorter residence time within the flow reactor compared to DRO and OZ. Simultaneously, the flow configuration enhances the mass transfer of the drug within the reactor through convection, thereby increasing the likelihood of collisions between EE2 and the freely diffusing reactive oxygen species generated by the photocatalyst. Consistent with findings from batch experiments, DRO exhibits the highest degradation rate constant, leading to near-complete depletion from the test solution within 450 min. In the flow system, a WO_3_-based photocatalyst operates under 400 nm irradiation, a condition under which it has already been proved that WO_3_ exhibits higher degradation efficiency of organic compounds with respect to other largely employed photocatalysts such as TiO_2_ [47]. The comparison of the results obtained in this study with the literature data is complicated due to the presence of numerous factors that can influence reactor performance [48]. Among these factors, the type of photocatalyst is particularly significant. Photocatalytic activity is influenced not only by chemical composition but also by structural characteristics, dimensions, surface impurities, and the surface area. Only a few studies have been carried out to investigate the efficiency of WO_3_ in the photodegradation of the contaminants of emerging concern in a continuous flow reactor. Among them, Martins [44] had reported a transformation rate constant of 3.5 × 10^−2^ min^−1^ and 2.2 × 10^−2^ min^−1^ for tetracycline, obtained from two photoreactors having different configurations, which are of the same order of magnitude as those obtained in the present study.

### 3.4. Characterization of the Degradation Intermediates

After 8 h of circulation in the flow system, the EE2, DRO and OZ samples were analyzed by LC/MS in positive (DRO and OZ) and negative (EE2) ion modes. MS/MS data were acquired in order to establish the best ion transitions for use in MRM analysis. The instrument settings were adjusted to maximize the response of each precursor–product combination. Chromatographic separation of the compounds was achieved on a concentration gradient reversed-phase column with a total run time of 15 min at a flow rate of 150 µL/min. The chromatograms of the different drugs were acquired by monitoring the *m*/*z* transitions 295 > 145 and 295 > 143 for the [EE2 − H]^−^ ion. The [DRO + H^+^]^+^ ion was monitored via the *m*/*z* transitions 367 > 97 and 367 > 91 and the [OZ + H^+^]^+^ ion via the *m*/*z* transitions 287 > 269 and 287 > 51. As mentioned earlier, the degradation of pharmaceuticals follows a scheme involving the reaction of contaminants with hydroxyl radicals (OH•), whose presence has been confirmed through EPR measurements. A general scheme is reported in Supplementary Information (Scheme S1). In the following, the degradation intermediates originating from the oxidation of the three drugs investigated are described.

**Scheme 1:**
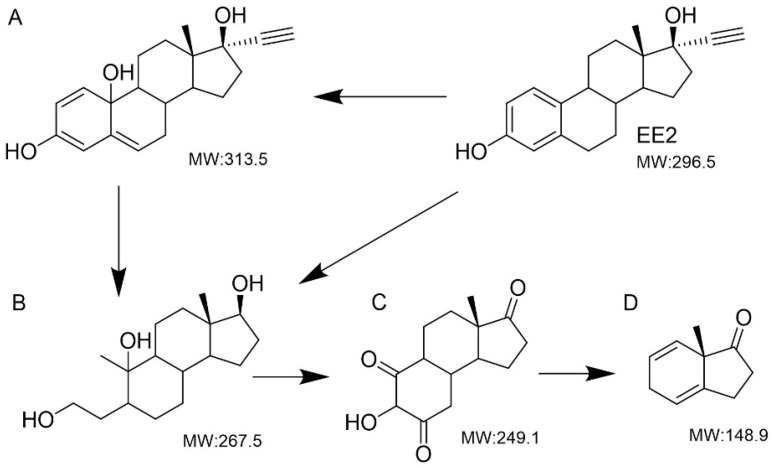
Proposed EE2 degradation pathway and intermediates structures (**A**–**D**) according to LC/MS analysis.

Mass spectrometry data show that EE2 was transformed into intermediate products of smaller molecular weight. To effectively track the conversion, it was necessary to identify the intermediates and by-products. The mass spectra show four characteristic peaks; the precursor ions detected in the sample were at *m*/*z* of 313, 267, 249, and 148. Compared with other studies on the photo-transformation products of EE2, it is found that the conversion of EE2 by HO• attack can occur, generating the by-products reported in Scheme 1 [49,50], according to a pathway consistent with the known reactivity of WO_3_ upon band gap excitation in the presence of aqueous electrolytes. Reactive oxygen species like HO• are strong enough to break the C-C, C-O, and O-H bonds in the EE2 molecule and its intermediates. The overall photocatalytic degradation process of EE2 via WO_3_/st can be expressed as proposed in Scheme 1. HPLC/MS analysis confirms that, indeed, OH radicals are the main reactive oxidants in the photodegradation of EE2 in the presence of WO_3_/st due to the presence of hydroxylated intermediates. Intermediate A may correspond to a hydroxylated compound following the docking of OH with the addition of a hydroxyl group, linked to the benzene ring. Product B could be formed through processes such as oxidation, carbonyl, and ring breakdown. Product B, undergoing a sequence of oxidative attacks, could produce the oxygen-rich product C. The latter, after the complete oxidation and cleavage of the partly oxidized six-membered ring-bearing carbonyl and hydroxyl groups, affords the molecular ion D seen at *m*/*z* 148 (see Table S1).

**Scheme 2:**
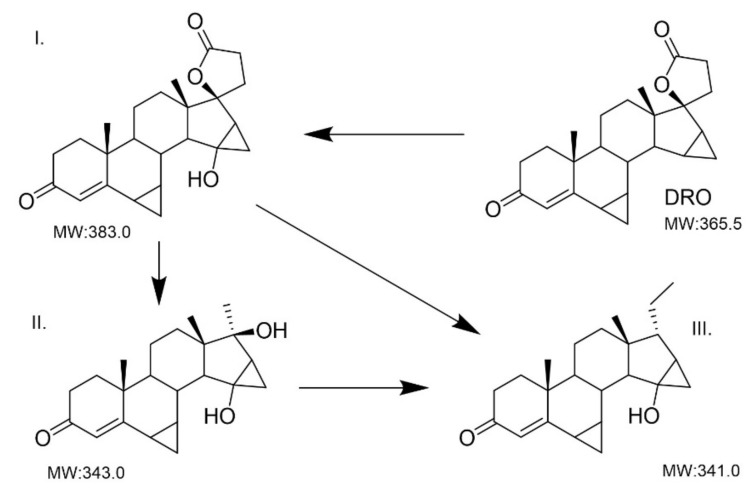
DRO degradation pathway and proposed intermediate structures (**I**–**III**) according to LC/MS analysis.

The photodegradation of DRO leads to the appearance of 383 *m*/*z*, 343 *m*/*z* and 341 *m*/*z* peaks (see Scheme 2 and Table S2). The 383 *m*/*z* by-product could be due to the addition of OH radicals onto the five-membered ring, similar to what was reported by Huang et al. [51] where the progesterone molecule (progenitor of the class to which DRO belongs) was studied. Subsequently, the oxidation process leads to the formation of other by-products, shorter than the starting molecules II and III.

**Scheme 3:**
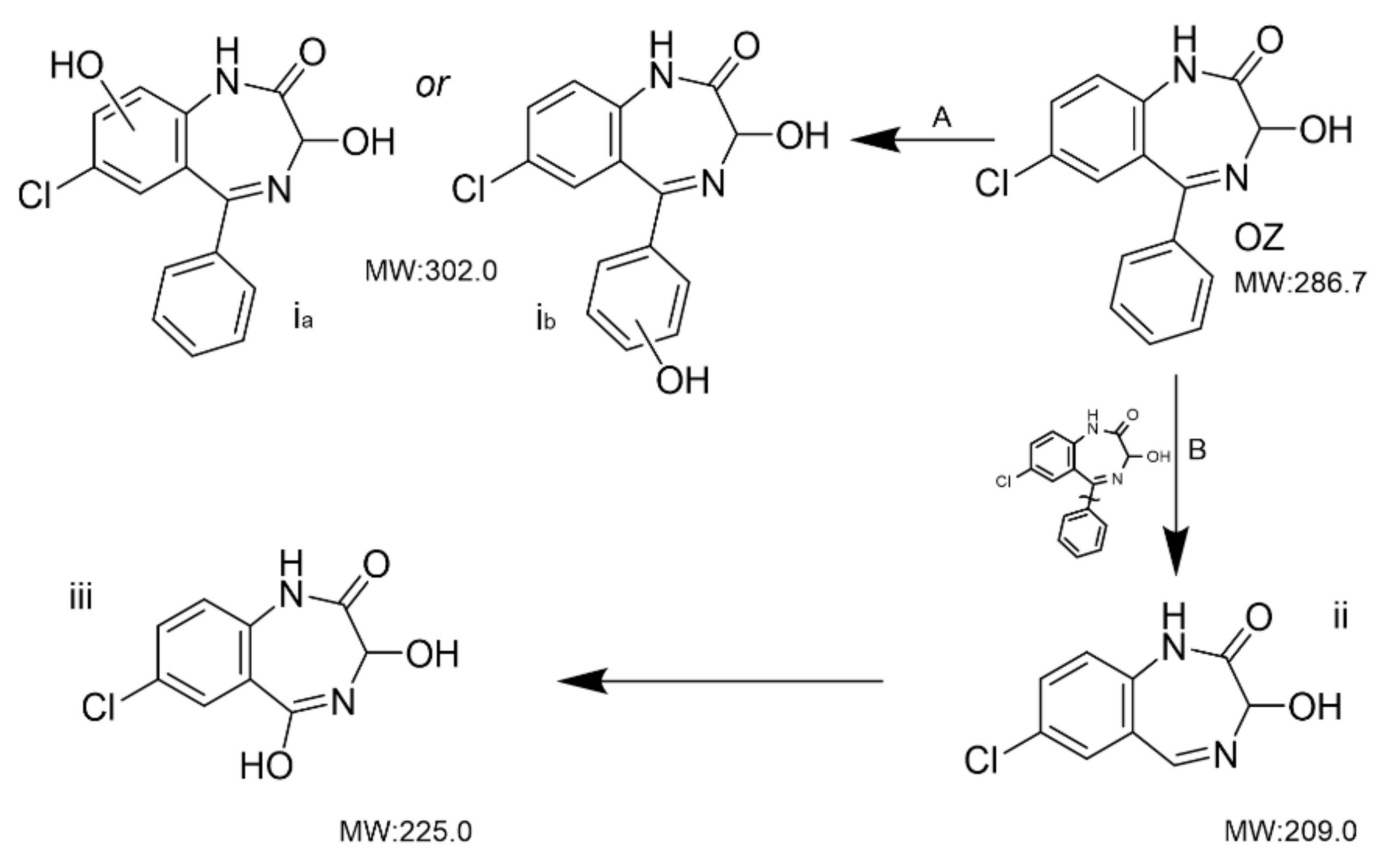
Proposed OZ degradation pathways and proposed by-product structures (**i_a_**,**i_b_**–**iii**) according to LC/MS analysis.

HPLC/MS analyses were also carried out to study the photodegradation intermediates of the oxazepam molecule (see Table S3). Four by-products were identified which correspond to 302 *m*/*z* (two possible structures), 225 *m*/*z* and 209 *m*/*z* peaks. The possible degradation pathway and photoproduced intermediates are shown in Scheme 3. As reported in other studies [52,53,54], where molecules such as alprazolam, which has similar structural characteristics as OZ and also belongs to the same benzodiazepine class, are investigated, it is hypothesized that the degradation of OZ may be mediated by photoproduced OH radicals as well. 

From the HPLC/MS analysis, hydroxylated intermediates were, in fact, identified, indicating the attack of OH radicals (pathway A) on the aromatic ring to form the products ia and ib. 

In pathway B, the oxazepam might undergo the cleavage of the bond between the nitrogen-containing heterocycle and the benzene ring, leading to detachment of the phenyl group (ii). Then, intermediate ii could be hydroxylated to product iii by further attack of OH radicals.

## 4. Conclusions

This work has been focused on the application of oriented WO_3_ nanostructures with high photoelectrochemical activity to water remediation, exploiting advanced photocatalytic oxidation processes. Oxidative degradation of pharmaceuticals, selected among steroid hormones and psychoactive drugs classified as high-priority emerging environmental contaminants, was successfully accomplished both in batch and flow photoreactors, with the latter exploiting easy-to-handle inert glass supports coated with solvothermally grown WO_3_ (WO_3_/st-GS). HPLC-MS analysis allowed for the separation and the clear identification of oxidation intermediates, pointing out that photogenerated HO radicals, generated upon the band gap excitation of WO_3_, are the primary agents for the advanced oxidative degradation of all the selected pharmaceutical targets. The study of the degradation kinetics has shown a different rate of drug degradation in the batch reactor compared to the flow one. In two out of three cases, the batch system displayed faster kinetics, probably due to a more efficient contact between the solution and the photocatalyst. In fact, dispersing the catalyst in the form of a fine powder leads to a greater interfacial area, resulting in a more efficient production of OH radicals. Conversely, in the flow system, WO_3_/st is immobilized on a solid support composed of millimeter-sized glass spheres, resulting in a decreased active surface/volume ratio and a less intimate contact with the solution carrying the drugs. This may result in the need for a longer treatment, particularly with robust molecules characterized by high chemical stability, like the family of benzodiazepines. Nevertheless, supported WO_3_ opens to easy handling and recovery of the photocatalyst and to the possibility of engineering flow systems suitable to treat larger volumes of contaminated water in a continuous fashion and with low counterpressure, while keeping the photocatalyst confined on a solid matrix.

## Figures and Tables

**Table 1:** Quantitative phase analysis and unit-cell parameters, as well as the crystallite size of the main phases, for the investigated samples. The R-weighted pattern refinement agreement factor was Rwp = 0.030 and 0.032 for the sample WO_3_/st and WO_3_/st-washed, respectively.

				Unit-Cell Parameters and Crystallite Size					
	Phase	QPA ^1^ Wt%	Space Group	a (Å)	b (Å)	c (Å)	β (°)	V (Å3)	Crystallite Size (nm)
WO_3_	WO_3_ (monoclinic)	96.0 (3)	P2_1_/n	7.3055 (8)	7.5256 (9)	7.6761 (10)	90.43 (1)	422.01 (9)	23 (1)
	WO_3_ (hexagonal)	4.0 (3)	P6_3_/mcm	7.354 (2)	–	7.397 (6)	–	346.5 (4)	–
WO_3_ washed	WO_3_ (monoclinic)	99.0 (2)	P2_1_/n	7.3106 (8)	7.5271 (9)	7.6813 (10)	89.97 (1)	422.69 (9)	21 (1)
	WO_3_ (hexagonal)	1.0 (2)	P6_3_/mcm	–	–	–	–	–	–

^1^ Quantitative Phase Analysis.

**Table 2:** Parameters obtained by non-linear fitting using a first-order kinetic model. The error is given as confidence interval at 95% of probability.

		k _PFO_ (min^−1^)	R^2^
Batch	EE2 WO_3_/st	0.0018 ± 0.0002	0.9778
	DRO WO_3_/st	0.0293 ± 0.0040	0.9969
	OZ WO_3_/st	0.0071 ± 0.0010	0.9936
Flow	EE2 WO_3_/st-GS	0.0019 ± 0.0002	0.9313
	DRO WO_3_/st-GS	0.0098 ± 0.0009	0.9880
	OZ WO_3_/st-GS	0.0022 ± 0.0002	0.9694

## Data Availability

Data are contained within the article.

## Supplementary Materials

The following supporting information can be downloaded at: https://www.mdpi.com/article/10.3390/nano14100860/s1, Figure S1: Cyclic voltammetry of FTO, FTO-TiO_x_ and FTO-TiO_x_-WO_3_ seed layer in 0.01 M K_4_FeCN_6_ and 0.1 M LiClO_4_; Figure S2: SEM image of WO_3_/coll; Figure S3: (a) Solar Simulator equipped with a AM1.5G filter and 380 nm UV cut-off filter, (b) 4 µg/mL drug solution in vial under magnetic stirring; Figure S4: WO_3_ films solvothermally grown on FTO before (a) and after (b) the annealing process. Electrodes equipped with the TiO_x_ adhesion layer are indicated by (Ti) (i.e., WO_3_/st-Ti-FTO); Figure S5: SEM image of WO_3_/st; Figure S6: EDS spectra of WO_3_/st; Figure S7: Phase identification for WO3 specimens grown on a microscope slide surface. The powder of both samples is mainly composed of WO_3_ in its monoclinic form (P2_1_/n; green reflections). WO_3_ in the hexagonal form (P6_3_/mcm; purple reflections) is also detected as associated minor phase; Figure S8: Rietveld refinement plots of the powder diffraction pattern for the WO_3_/st specimens’ growth on the microscope slide surface. The experimental profile is represented by black dots and the best-fit refinement profile is the flow red line. The lower green curve is the weighted difference between observed and calculated patterns. Vertical ticks mark the position of reflections for the identified phases in the two specimens. Figure S9: Glass column filled with WO_3_/st-GS; Figure S10: Nyquist plots of WO_3_/coll (red) and WO_3_/st (black) photoelectrode recorded at 0.6 V vs. SCE in 0.7 M Na_2_SO_4_ under AM1.5G illumination. 10 mV sinusoidal perturbation in the range 20000-0.1 Hz; Figure S11: Average JV performance of a batch of WO_3_/st photoanodes (black line) compared to some of our best internal standards WO_3_/coll (green line). 0.7 M Na_2_SO_4_ under AM1.5G illumination; Figure S12: EPR spectrum obtained after few seconds photoirradiation (λ > 400 nm) of aqueous suspension of WO_3_ containing H_2_SO_4_ (0.1 M) and pbn (5 × 10^−2^ M); Figure S13: Tauc Plots of WO_3_/st according to the direct (A) and indirect (B) transitions; Figure S14: J/V chopped curves under AM 1.5G illumination of WO_3_ in 0.1 M of LiClO_4_ in ACN solution in presence of 0.1 M of DRO (A) 0.1 M of EE2 (B) and 0.1 M of OZ; Figure S15: Comparison test (in presence of WO_3_): DRO in dark (DRODARK WO_3_/st)), DRO irradiated (DRO WO_3_/st) and DRO in dark in batch system with spheres coated with WO_3_ (DRODARK WO_3_/st-GS); Figure S16: Transmission spectrum of WO_3_/st loaded on glass spheres ([T% (WO_3_/st-GS − T% GS)]) obtained with an integrating sphere in diffuse reflection mode along an optical path of 1 cm; Figure S17: Transmission spectra (1 cm optical path) of naked glass spheres GS, WO_3_ coated glass spheres (WO_3_/st-GS) and WO_3_/st suspension (3.5 mg/mL) obtained in diffuse reflection mode; Scheme S1: Scheme for degradation of pollutants by WO_3_; Table S1: EE2 degradation intermediates, retention times, fragment ions and proposed structures; Table S2: DRO degradation intermediates, retention times, fragment ions and proposed structures; Table S3: DRO degradation intermediates, retention times, fragment ions and proposed structures.
