# Supplementary material for: Solvothermally Grown Oriented WO3 Nanoflakes for the Photocatalytic Degradation of Pharmaceuticals in a Flow Reactor
Source: Nanomaterials (Basel). 2024 May 15;14(10):860. doi: 10.3390/nano14100860 (PMC11124514; doi:10.3390/nano14100860)
Supplement: Supplementary file 1 [file nanomaterials-14-00860-s001.zip › nanomaterials-2995449-supplementary.pdf]

Supplementary Material

Solvothermally Grown Oriented WO_3 Thin-film for the Photocatalytic Degradation of Pharmaceuticals in a Flow Reactor

Mirco Cescon [1], Claudia Stevanin [2], Matteo Ardit [3,5], Michele Orlandi [4], Annalisa Martucci [3], Tatiana Chenet [2], Luisa Pasti [2,*], Stefano Caramori [1a,*], Vito Cristino [1]

[1] Department of Chemical, Pharmaceutical and Agricultural Sciences, University of Ferrara, Via L. Borsari 46, Ferrara, 44121, Italy

a National Interuniversity Consortium of Materials Science and Technology (INSTM), University of Ferrara Research Unit, 44121 Ferrara, Italy

[2] Department of Environmental and Prevention Sciences, University of Ferrara, Via L. Borsari 46, Ferrara, 44121, Italy

[3] Department of Physics and Earth Sciences, University of Ferrara, Via Saragat 1, Ferrara, 44121, Italy

[4] Department of Physics, University of Trento, Via Sommarive 14, Trento, 38123, Italy

[5] Department of Geosciences, University of Padova, Via Gradenigo 6, IT-35131 Padova

* Correspondence: LP: luisa.pasti@unife.it; Tel.: +390532455346, SC: stefano.caramori@unife.it; Tel.: +390532455474

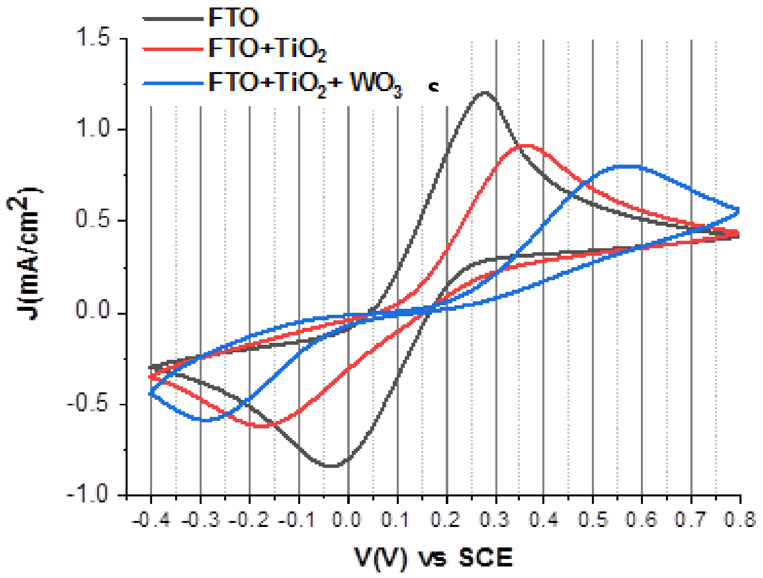

Figure S1. Cyclic voltammetry of FTO, FTO-TiO_x and FTO-TiO_x-WO_3 seed layer in 0.01 M K_4FeCN_6 and 0.1 M $LiClO_4$.

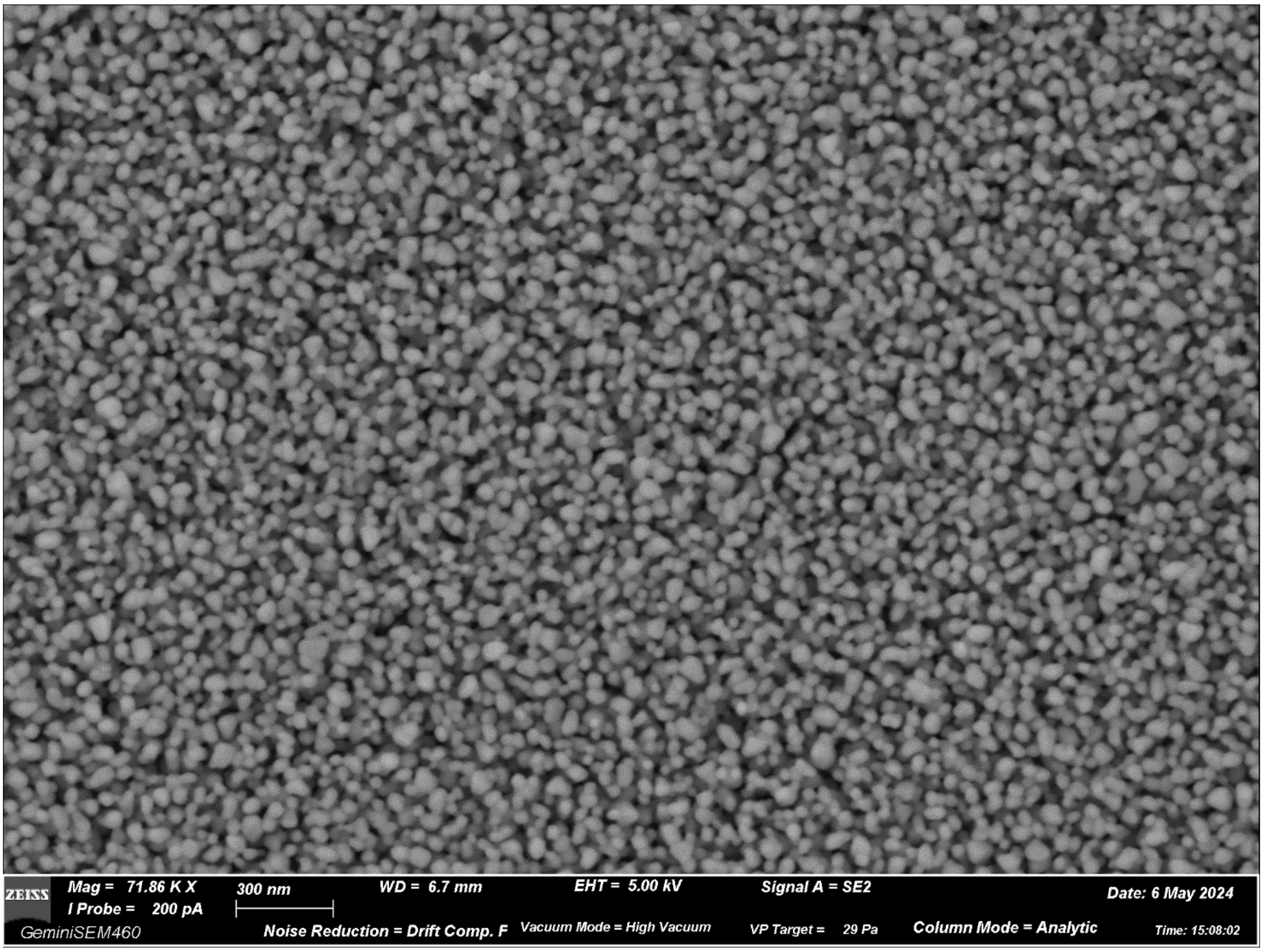

Figure S2. SEM image of WO_3/coll.

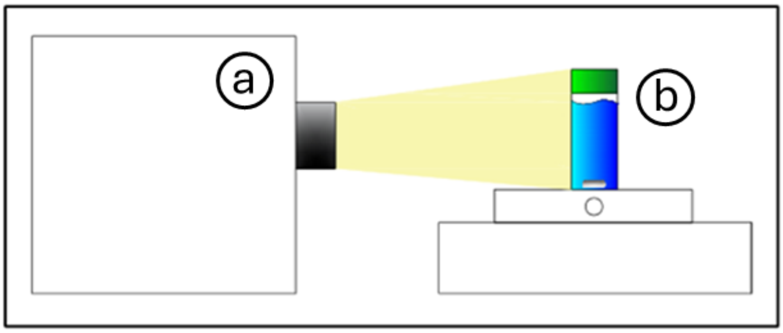

Figure S3. (a) Solar Simulator equipped with a AM1.5G filter and 380nm UV cut-off filter, (b) 4 μg/ml drug solution in vial under magnetic stirring.

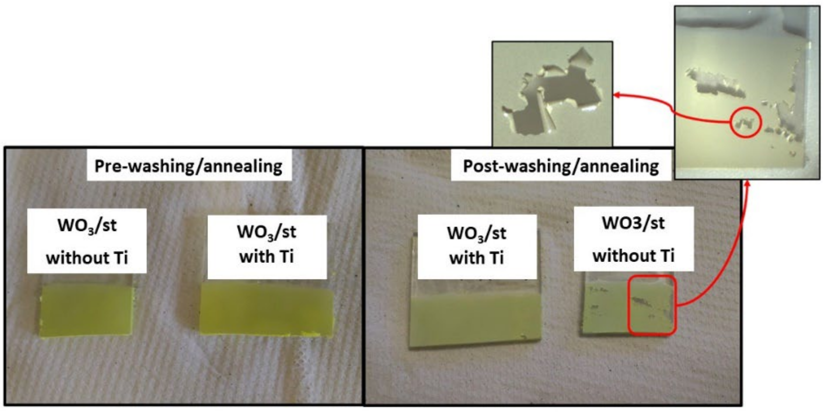

(a) **(b)**

Figure S4. WO_3 films solvothermally grown on FTO before (a) and after (b) the annealing process. Electrodes equipped with the TiO_x adhesion layer are indicated by (Ti) (i.e., WO_3/st-Ti-FTO).

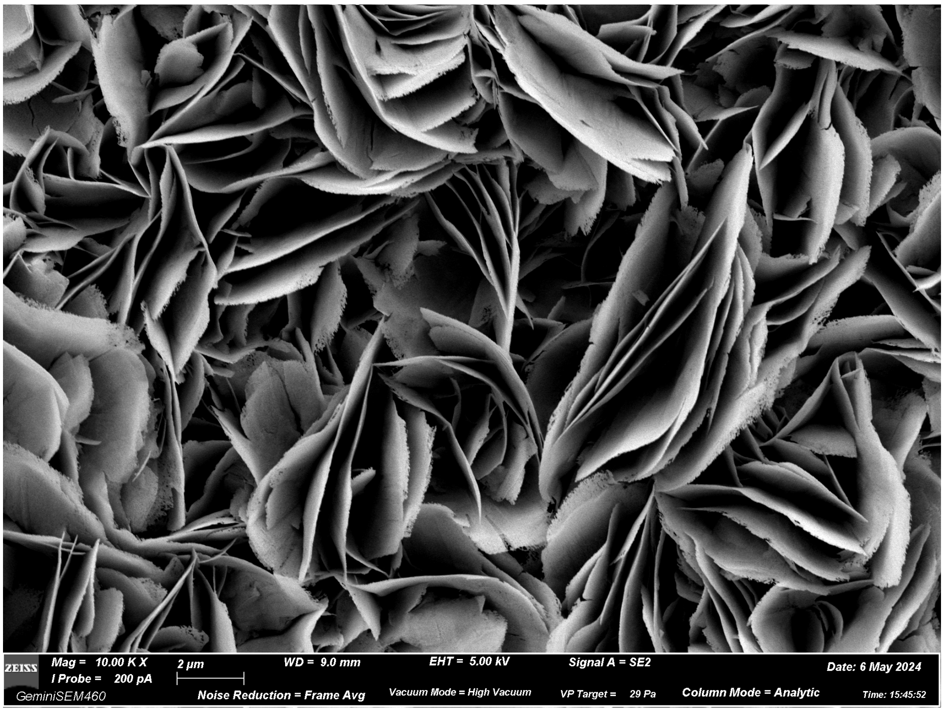

Figure S5. SEM image of WO_3/st.

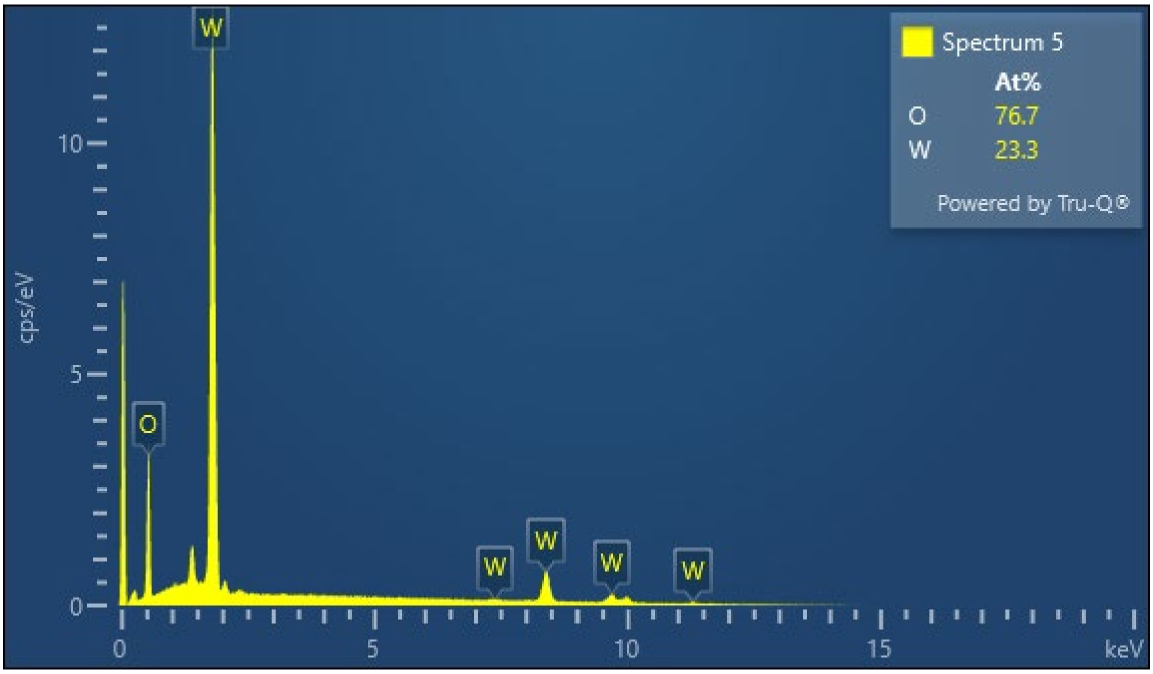

Figure S6. EDS spectra of WO_3/st.

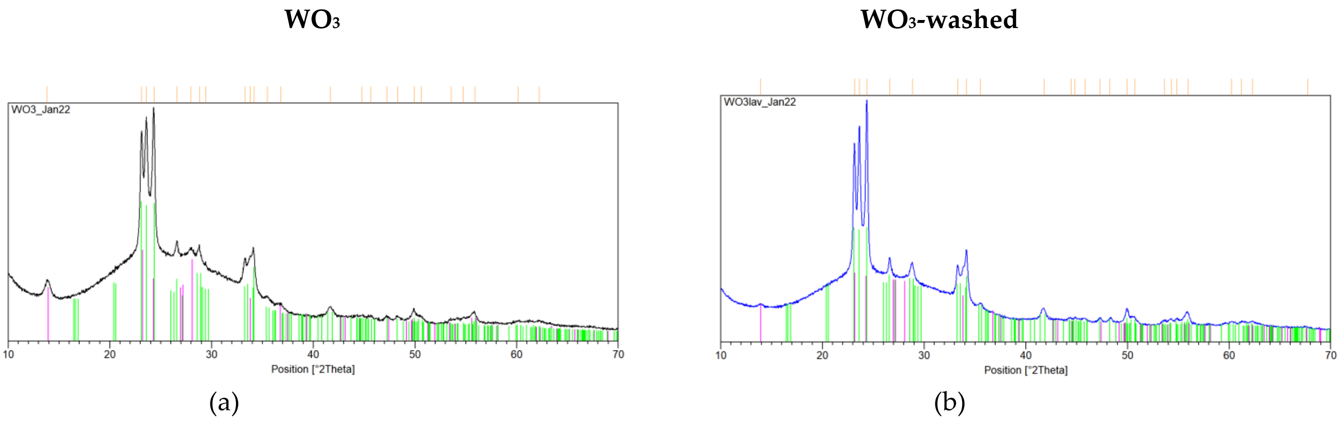

Figure S7. Phase identification for WO_3 specimens grown on a microscope slide surface. The powder of both samples is mainly composed by WO_3 in its monoclinic form ($P2_1/n$; green reflections). WO_3 in the hexagonal form ($P6_3/mcm$; purple reflections) is also detected as associated minor phase.

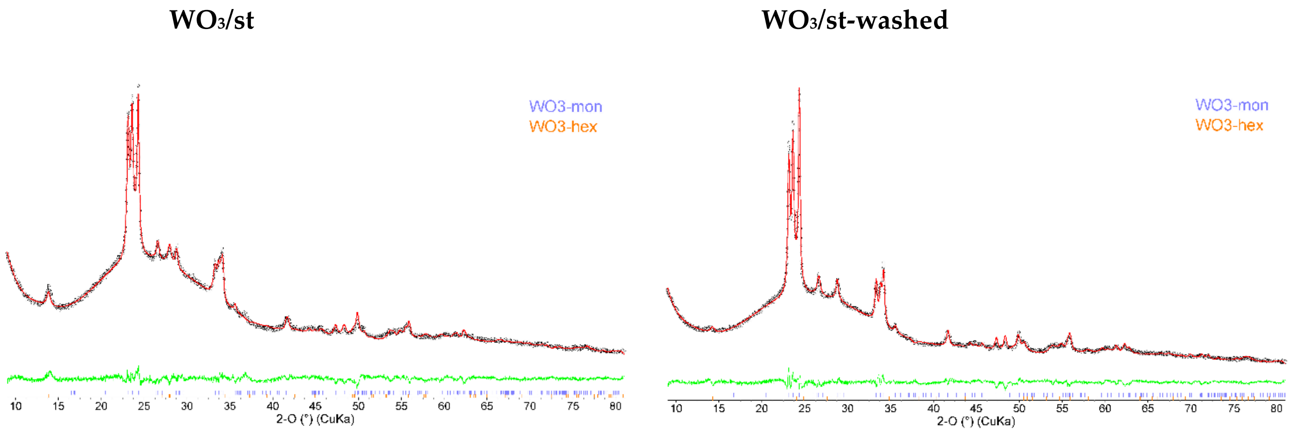

(a) (b)

Figure S8. Rietveld refinement plots of the powder diffraction pattern for the WO_3/st specimens growth on the microscope slide surface. The experimental profile is represented by black dots and the best-fit refinement profile is the flow red line. The lower green curve is the weighted difference between observed and calculated patterns. Vertical ticks mark the position of reflections for the identified phases in the two specimens.

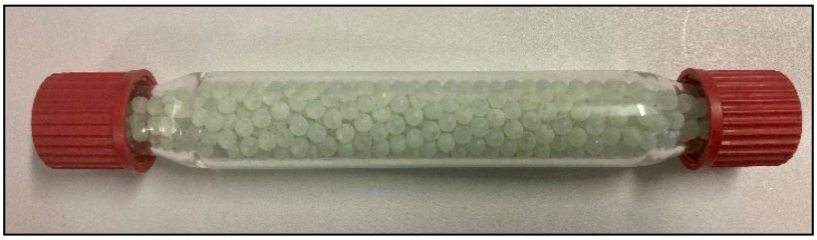

Figure S9. Glass column filled with WO3/st-GS.

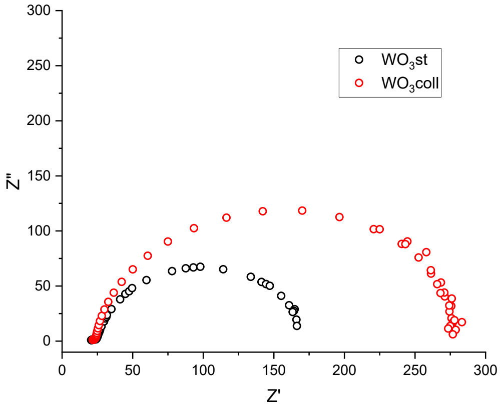

Figure S10. Nyquist plots of WO_3/coll (red) and WO_3/st (black) photoelectrode recorded at 0.6V vs SCE in 0.7M Na_2SO_4 under AM1.5G illumination. 10 mV sinusoidal perturbation in the range 20000-0.1 Hz.

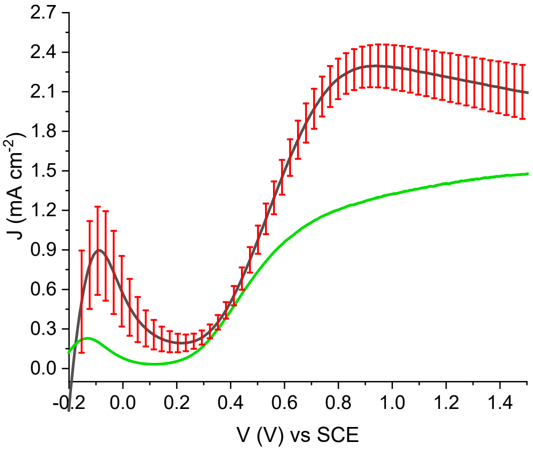

Figure S11. Average JV performance of a batch of WO_3/st photoanodes (black line) compared to some of our best internal standards WO_3/coll (green line). 0.7M Na_2SO_4 under AM1.5G illumination.

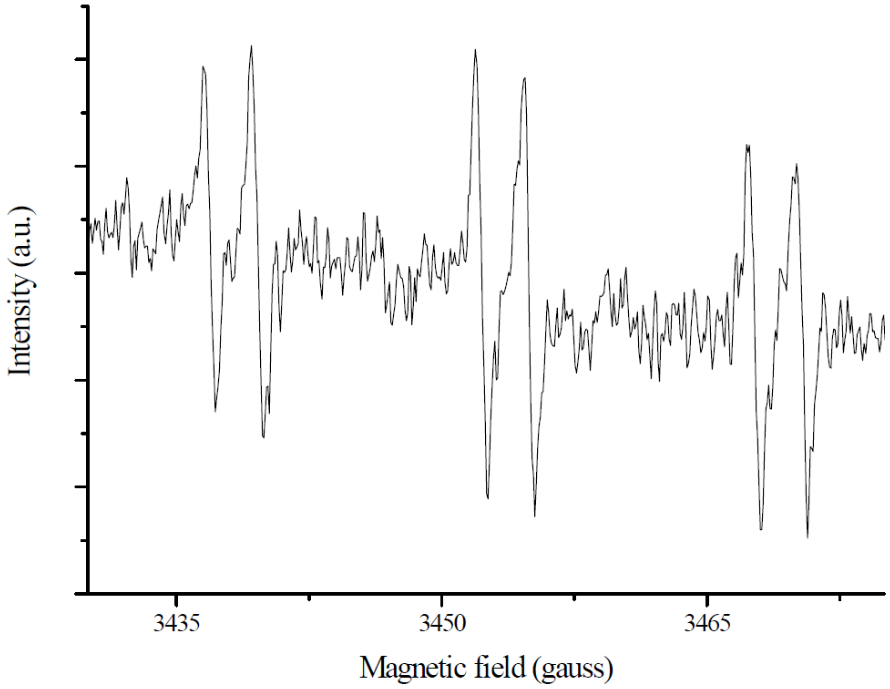

Figure S12. EPR spectrum obtained after few seconds photoirradiation (λ > 420 nm) of aqueous suspension of WO3 containing H_2SO_4 (0.1 M) and pbn (5×10^{-2} M).

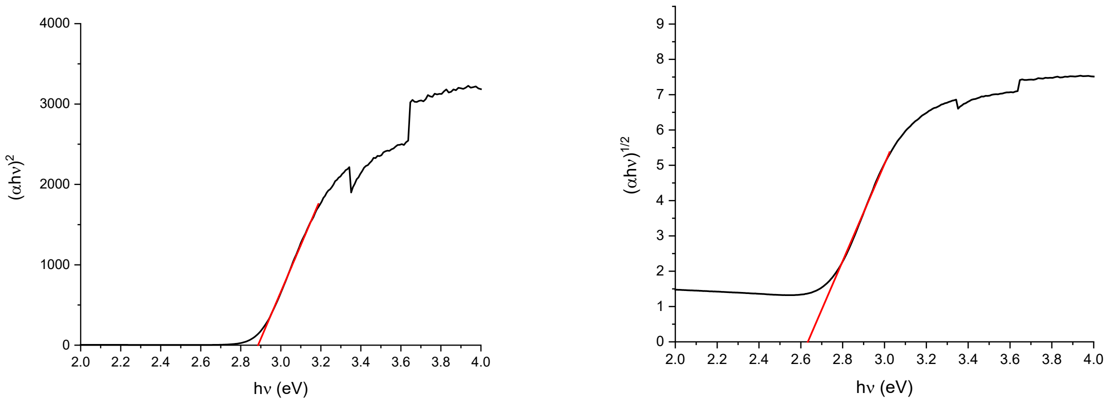

(A) (B)

Figure S13. Tauc Plots of WO_3/st according to the direct (A) and indirect (B) transitions.

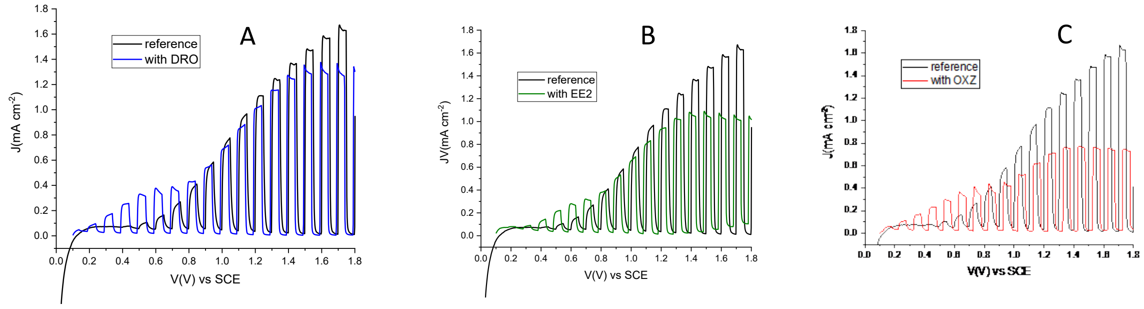

Figure S14. J/V chopped curves under AM 1.5G illumination of WO3 in 0.1M of LiClO4 in ACN solution in presence of 0.1M of DRO (A) 0.1M of EE2 (B) and 0.1M of OZ (C).

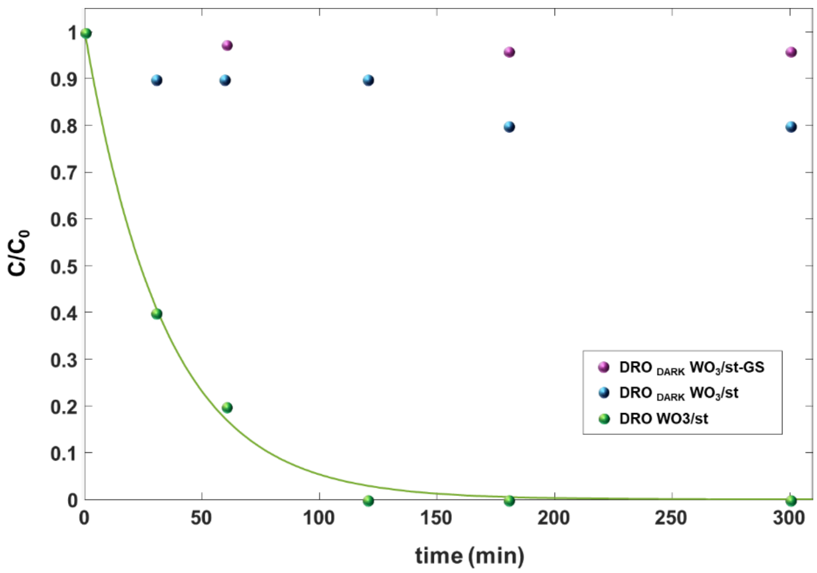

Figure S15. Comparison test (in presence of WO_3): DRO in dark (DRO_{DARK} WO_3/st)), DRO irradiated (DRO WO_3/st) and DRO in dark in batch system with spheres coated with WO_3 (DRO_{DARK} WO_3/st-GS).

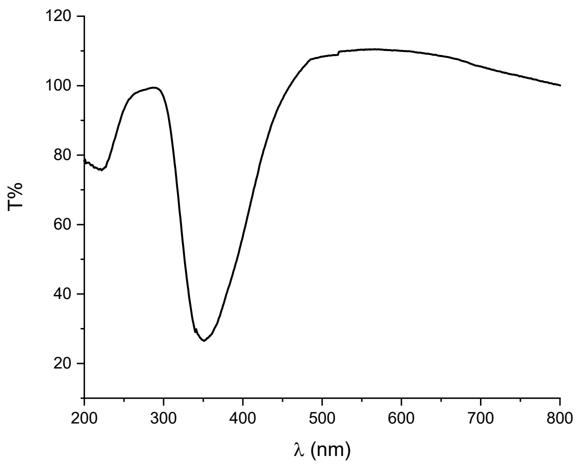

Figure S16. Transmission spectrum of WO_3/st loaded on glass spheres ([T % (WO_3/st-GS – T % GS)]) obtained with an integrating sphere in diffuse reflection mode along an optical path of 1 cm.

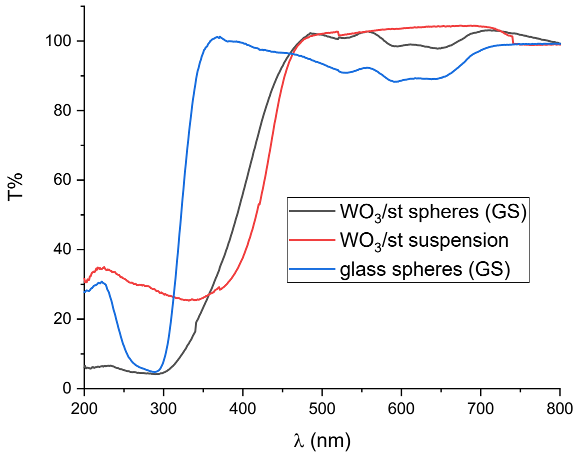

Figure S17. Transmission spectra (1 cm optical path) of naked glass spheres GS, WO_3 coated glass spheres (WO_3/st-GS) and WO_3st suspension (3.5 mg/ml) obtained in diffuse reflection mode.

$$WO_3 + h\nu \longrightarrow e^- + h^+ + WO_3$$

$$H_2O \longrightarrow H^+ + OH^-$$

$$OH^- + h^+ \longrightarrow \cdot OH$$

$$\cdot OH + \text{contaminant} \longrightarrow \text{oxidation or degradation of pollutant}$$

Scheme S1. Scheme for degradation of pollutants by WO3.

Table S1. EE2 degradation intermediates, retention times, fragment ions and proposed structures.

Precursor Ion (m/z)	*t_r (min)*	*MS^2 (m/z)*	*Structure*
148.9	*6.52*	*133.9*	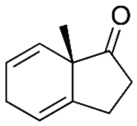
249.1	*9.56*	*231.1,138.1*	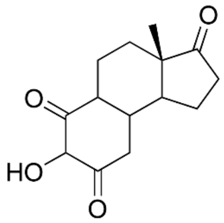
267.5	*12.07*	*220.5, 252.5*	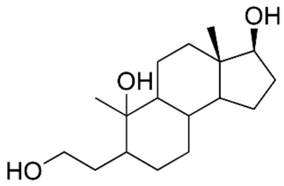
313.5	*14.10*	*288.5, 295.5*	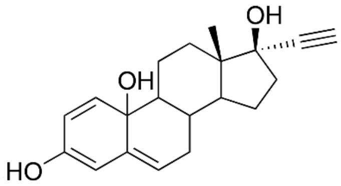

Table S2. DRO degradation intermediates, retention times, fragment ions and proposed structures.

Precursor Ion (m/z)	*t_r (min)*	*MS^2 (m/z)*	*Structure*
341.0	*7.02*	*192.0; 149*	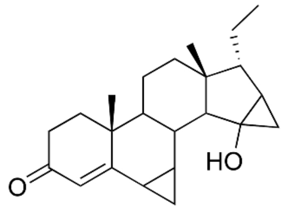
343.0	*7.53*	*195.0;151*	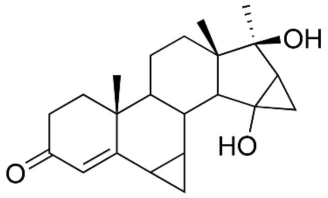
383.0	*13.06*	*310;190*	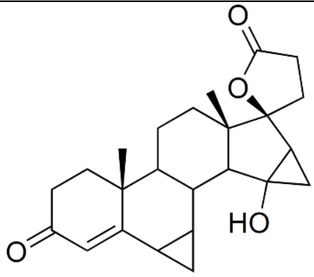

Table S3. OZ degradation intermediates, retention times, fragment ions and proposed structures.

Precursor Ion (m/z)	*t_r (min)*	*MS^2 (m/z)*	*Structure*
209.0	*5.23*	*192.0; 130.0*	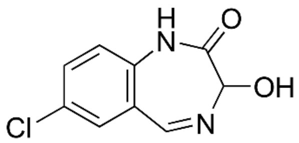
225.0	*6.01*	*208.0; 147.0*	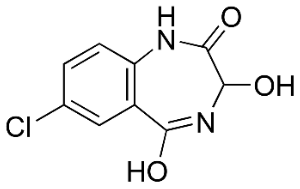
302.0	*7.29*	*287.0; 226.0; 192.0*	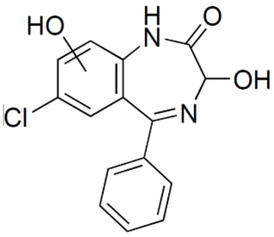 *or* 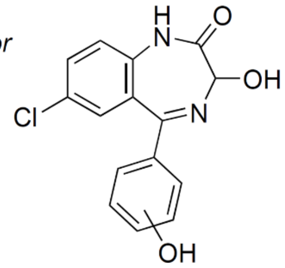
